# Oral intake of aripiprazole compromises male fertility in *Drosophila*

**DOI:** 10.1186/s13062-025-00698-9

**Published:** 2025-11-11

**Authors:** Amrita Mukherjee, James D. Hurcomb, Samantha H. Y. Loh, L. Miguel Martins

**Affiliations:** MRC Toxicology Unit, University of Cambridge, Gleeson Building, Tennis Court Road, Cambridge, CB2 1QR UK

**Keywords:** *Drosophila*, Testes, Spermatogenesis, Mitochondria, JNK signalling, Stem cells, Germ cells, Lysosomes, Phagocytosis

## Abstract

Antipsychotics have reported off-target effects, but their impact on subcellular organelles and cellular homeostasis in various organ systems is poorly understood. This study explored the off-target effects of aripiprazole on the male reproductive system using *Drosophila* as a model. Aripiprazole binds nonspecifically to mitochondrial complex I, and here we investigated the effect of an aripiprazole-containing diet on spermatogenesis. We showed that aripiprazole increases the level of mitochondrial reactive oxygen species (ROS) and disrupts the homeostasis of germ cell development in the testes. The cyst cells surrounding the spermatogonia showed an increase in JNK signalling, while there was enhanced LysoTracker staining of spermatogonial cysts and defects in the later stages of spermatid individualisation. Our results revealed a connection between mitochondrial complex I dysfunction and increased germ cell loss by lysosomal degradation, resulting in decreased fertility. We conclude that aripiprazole-induced mitochondrial toxicity in germ cells results in increased loss of spermatogonial cysts and defects in spermatogenesis. We showed that diets supplemented with antioxidants or the expression of mitochondrial superoxide dismutase in spermatogonial cells can alleviate excess mitochondrial ROS-induced defects.

**Supplementary information:**

The online version contains supplementary material available at 10.1186/s13062-025-00698-9.

## Introduction

Aripiprazole is a third-generation antipsychotic that crosses the blood‒brain barrier and interacts with dopamine D_2_ receptors [1, 2]. It acts as a partial agonist to these receptors with an affinity similar to that of dopamine but triggers a response that is lower than that of dopamine but higher than that of an antagonist (reviewed in [3]). Aripiprazole needs to engage more than 90% of D_2_ receptors to be clinically active. Despite this high occupancy, it has fewer side effects than other antipsychotics [4] and has frequently replaced first-generation antipsychotics (reviewed in [5]). Recently, we showed that aripiprazole can also bind and inhibit mitochondrial respiratory complex I (Complex I or NADH:ubiquinone oxidoreductase I) [6]. However, the consequences of this off-target effect of aripiprazole on different organ systems are unknown.

Male mice injected with aripiprazole accumulate this drug in the brain and several other organs [7]. A study assessing the transport of aripiprazole between the blood and different tissues revealed that the concentration of aripiprazole in the brain was approximately three times greater than that in the plasma. However, this study also reported that the quantity of aripiprazole present in the testes of male mice exceeds that observed in the brain [7]. Two independent morphological and ultrastructural studies of testes in prepubertal and adult rats orally administered aripiprazole revealed a generalised loss of multiple stages of germline cells, increased presence of vacuoles in somatic Sertoli cells, increased intercellular gaps within the testes, and a loss of normal cellular architecture [8, 9].

Aripiprazole in humans can achieve blood concentrations between 0.1 to 1 µM (reviewed in [10]). At therapeutic concentrations, aripiprazole binds strongly to serum albumin proteins [11, 12] and has a high steady-state volume distribution that indicates the drug’s presence in the extravascular system [13]. *Drosophila* kept on an aripiprazole-supplemented diet accumulated the drug at concentrations ranging from 0.10 µM to 1.7 µM [14]. Flies kept on an aripiprazole-containing diet had mitochondrial damage in brain and muscle tissues [6] and disruption of gastrointestinal system [14], indicating that the drug can access multiple tissues in this model. The testes are male organs responsible for making sperm and are also involved in the production of male-specific hormones. While there are significant differences in the specific organisation of the testis between *Drosophila* and vertebrates, *Drosophila* is an excellent model for studying sperm development and male fertility because of the conservation of key molecular mechanisms and cellular processes involved in spermatogenesis across species. The high degree of evolutionary conservation in proteins and signalling pathways, such as those involved in mitochondrial function and transcriptional regulation, allows insights gained from *Drosophila* studies to be applicable to vertebrates, including humans (reviewed in [15]). *Drosophila* testes are easily dissected, allowing visualisation of spermatogenesis stages (reviewed in [16]). Additionally, the genetic tractability and well-characterised biology of *Drosophila* mean that this insect is a robust and well-established model for the study of spermatogenesis (reviewed in [17]).

**Fig. 1:**
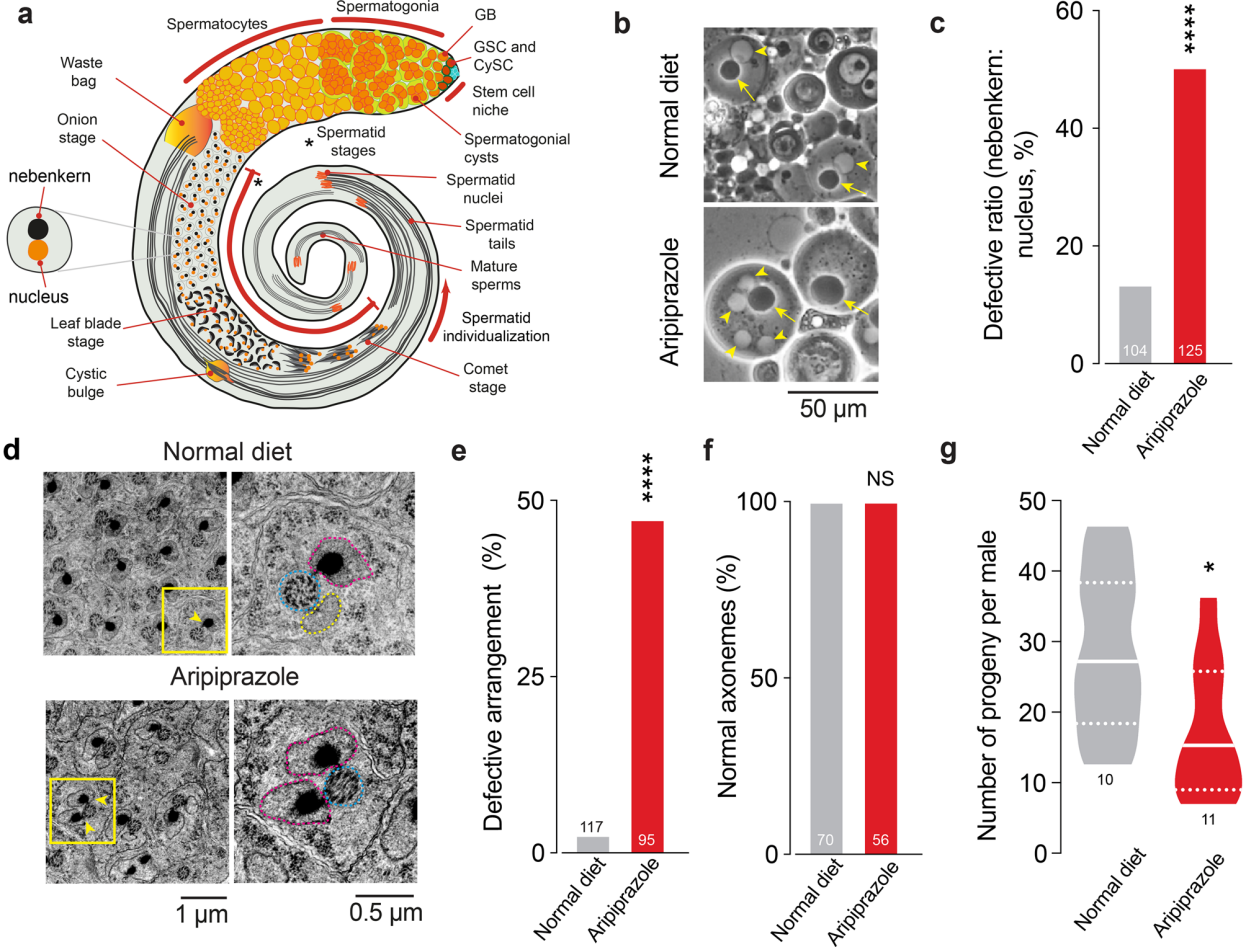
An aripiprazole-supplemented diet results in defects in spermatogenesis. (**a**) A schematic drawing of the anatomy of an adult *Drosophila* testis showing the different stages of spermatogenesis with germline stem cells (red), GSC; cyst stem cells (dark green), CySCs; hub cells (blue); somatic cyst cells (light green); spermatogonial cysts (dark orange) and spermatocytes (light orange) (**b**) Representative images of mitochondrial defects observed in post-meiotic spermatids. The ‘onion-stage’ spermatids contain one haploid nucleus (yellow arrowhead) and one mitochondrial derivative (nebenkern, yellow arrow) in a 1:1 ratio of roughly equal diameter. In flies kept on an aripiprazole-supplemented diet, this ratio is altered, and quantification (**c**) of this ratio shows the percentage of spermatids with a defective ratio (asterisks; Fisher’s exact test; n indicates the number of analysed round spermatids). (**d**) Ultrastructural analysis of spermatid cysts after individualisation where each axoneme (dotted outline in blue) is associated with a major (dotted outline in purple) and a minor (dotted outline in yellow) mitochondrial derivative. The yellow arrowheads indicate the dark crystalline structure in the major mitochondrial derivative. The normal arrangement of 1(axoneme):1(major mitochondrial derivative):1(minor mitochondrial derivative) is perturbed in flies kept on an aripiprazole-containing diet. The panels on the right show high-magnification images of the yellow boxed regions. (**e**) Quantification of the percentage of spermatids showing defects in this arrangement (asterisks; Fisher’s exact test; n indicates the number of elongated individualised spermatids analysed). (**f**) Quantification of the normal (9+2) arrangement of microtubules in axonemes in individualised spermatids (NS, Fisher’s exact test; n indicates the number of elongated individualised spermatids analysed). (**g**) Male flies kept on an aripiprazole supplemented diet produced fewer offspring than those kept on normal diet (asterisks; unpaired t-test; n represents the number of males). The analysis results from crosses between males kept on an aripiprazole-supplemented or normal diet for 11 days and 2–3-day-old virgin *w*^*1118*^ females maintained on a standard diet. Genotypes (**b-g**): *w*^*1118*^*CS*

Germ cells undergo spermatogenesis to transform into haploid spermatozoa. The process begins when stem cells undergo asymmetric mitotic division. The stem cells (both germline and cyst stem cells) are present at the tips of the testes in contact with the ‘hub’. Somatic hub cells together, but independently, with cyst stem cells (CySCs) help maintain the stem cell state of the germ cells (reviewed in [18]). Each germline stem cell (GSC) divides asymmetrically to give rise to a gonioblast (GB) (reviewed in [19]). Each GB undergoes 4 rounds of mitotic division to give rise to 16 precursor cells called spermatogonia. The spermatogonial stages (comprising spermatogonial cysts) carry either 2, 4, 8 or 16 cells. The cells within each cyst are interconnected by cytoplasmic bridges and a branched organelle called the fusome. The fusome in male testes allows synchronous death of a spermatogonial cyst in response to DNA damage [20]. After mitosis, once at the 16-cell stage, mitochondria undergo structural changes as the spermatocytes mature. As the primary spermatocytes enter the growth phase, a ball-like structure called a ‘mitoball’ is formed, composed of aggregated mitochondria [21]. The spermatocytes increase in size 25-fold and then undergo meiosis to give rise to 64 round spermatids (Fig. 1a). In the round spermatid stage (also called the onion stage), the mitochondria transform into a spherical structure called the nebenkern. The nebenkern unfurls into the major and minor mitochondrial derivatives that stretch and elongate as the round spermatids morph into the ‘leaf blade’ and ‘comet’ stages (Fig. 1a and reviewed in [22]). These mitochondrial derivatives are associated with the axoneme, a microtubule-based cytoskeletal structure arranged in a 9 + 2 arrangement that forms part of the final sperm tail. In mature spermatids, the axoneme and the two mitochondrial derivatives run along the length of the sperm tails. More mature sperm are found towards the base of the testes, and they are ultimately deposited in the seminal vesicles (Fig. 1a).

Mitochondria provide the energy required for sperm cells to move and fertilise eggs, which is essential for successful sexual reproduction. Stem cells are regulated by local signals generated at the stem cell niche but are strongly influenced by the presence of drugs, nutrients and metabolites. Mitochondria are at the heart of cellular metabolism and have the capacity to integrate both intrinsic and extrinsic signals to regulate stem cell maintenance, differentiation and death. Mitochondrial dynamics, mitophagy, and mitochondrial respiration all contribute to various stages of spermatogenesis to produce viable sperm cells (reviewed in [22]).

Because aripiprazole accumulates in the testis [7] and has off-target effects on mitochondria [6], we explored the consequences of an aripiprazole-supplemented diet on the fertility of male flies. When aripiprazole is used to treat patients with schizophrenia, it is typically administered chronically via the oral route [23]. We modelled the chronic administration of aripiprazole in flies to understand the long term side-effects of this drug on spermatogenesis. We show that the ingestion of aripiprazole disrupts spermatogenesis and compromises male fertility. We found that flies kept on a diet containing aripiprazole exhibit a loss of spermatogonial cells via lysosomal degradation, with downstream consequences for spermatid individualisation and fertility. Overall, by exploring the adverse effects of the drug aripiprazole on spermatogenesis, our study demonstrated the critical role of functional mitochondrial complex I (CI) and ROS in spermatogonial survival and male fertility.

## Results

### An aripiprazole-supplemented diet compromises fly spermatogenesis and fertility without affecting courtship behaviour

We decided to model the potential toxicity of aripiprazole in the male reproductive system of *Drosophila* by first determining whether flies maintained on an aripiprazole-containing diet have defects in spermatogenesis. To test this, we kept freshly hatched control male flies on a diet supplemented with 1 mM aripiprazole. We checked for abnormalities in the post meiotic stages of spermatogenesis, where the mitochondria undergo a dramatic transformation (reviewed in [22]. We have previously shown that mitochondrial dysfunction has an effect on these stages [24]. In the round spermatid stage, mitochondria come together to form a ball-like structure called the nebenkern (Fig. 1a). The nebenkern and the nuclei at this stage are roughly equal in size and are present at a 1:1 ratio (Fig. 1a, b). We found that this arrangement was perturbed in the testes of flies supplemented with an aripiprazole-containing diet (Fig. 1b, c). We next examined elongating spermatids at the post-individualisation stages (Fig. 1a and d). At this stage, each spermatid consists of a microtubular structure, the axoneme, with a 9 + 2 arrangement of microtubule filaments (reviewed in [25]. At this stage, the spermatids also contain two mitochondrial derivatives, a major and a minor mitochondrial derivative (Fig. 1d, yellow boxed region and yellow arrowheads). We performed an ultrastructural analysis of fly testes and found that flies kept on an aripiprazole-containing diet presented defects in the arrangement of 1 axoneme:1 minor mitochondria:1 major mitochondrial derivative (Fig. 1d, e), but there were no changes in the 9 + 2 arrangement of microtubules in the axoneme (Fig. 1f). To test whether these defects in spermatogenesis are associated with fertility defects, we crossed male flies kept on an aripiprazole-supplemented diet with virgin females maintained on standard diet. We found that males kept on a diet supplemented with aripiprazole for 11 days crossed with control virgin females, kept on standard diet, presented a significant reduction in the number of offspring (Fig. 1g). Together, these data show that the exposure of adult *Drosophila* males to aripiprazole results in defects in spermatid maturation and a reduction in fertility. Next, because an aripiprazole-supplemented diet could alter the feeding patterns of flies kept on this diet, and also because starvation affects spermatogenesis [26, 27], we used fly body weight as a surrogate marker of their food intake. We measured the body weight of adult males kept on a normal diet or an aripiprazole-supplemented diet during their lifespan. On average, the body weight of flies kept on normal food was approximately 0.7 mg across our measurements and was not altered when flies were kept on an aripiprazole-supplemented diet (Supplementary Fig. 1a). We conclude that the reduction in fertility in males kept on a diet supplemented with aripiprazole is not due to nutritional deficiency.

Dopaminergic signalling in the blood brain barrier cells of *Drosophila* mediates courtship behaviours [28]. Because aripiprazole is a partial agonist of the D2 dopamine receptor, and modulates dopamine signalling, we examined the courtship behaviours of male flies kept on an aripiprazole-supplemented diet. We did not find any alterations in the courtship behaviours of male flies kept on an aripiprazole-supplemented diet, (Supplementary Fig. 1b and c) nor any changes in their courtship index, or latency (Supplementary Fig. 1d and e). We conclude that an aripiprazole-supplemented diet does not impact courtship rituals in male flies.

### An aripiprazole-supplemented diet decreases mitochondrial function and disrupts mitochondrial structure in spermatogonia

**Fig. 2:**
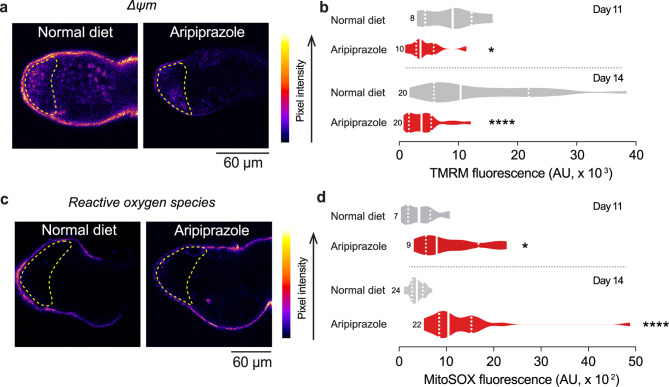
Flies kept on an aripiprazole-supplemented diet have impaired mitochondrial function and increased levels of reactive oxygen species in the germarium. (**a** and **b**) Aripiprazole supplementation reduces Δψm in the germarium. The area analysed is indicated by yellow dotted lines. Representative confocal images (**a**), showing TMRM intensity levels mapped using the ‘fire’ lookup table in FIJI. Quantification (**b**) of TMRM intensity in 11- and 14-day-old flies (asterisks; mann-whitney test; n indicates the number of analysed testes; AU, arbitrary units). (**c** and **d**) Increased levels of mitochondrial reactive oxygen species (mtROS) were detected in the spermatogonia of flies kept on an aripiprazole-containing diet. The area analysed is indicated by yellow dotted lines. Representative confocal images (**c**), showing MitoSOX intensity levels mapped using the ‘fire’ lookup table in FIJI. Quantification (**d**) of MitoSOX intensity levels in 11- (asterisk; unpaired t-test; n indicates the number of analysed testes) and 14-day-old (asterisks; mann-whitney test; n indicates the number of analysed testes; AU, arbitrary units). Genotypes (**a-d**): *w*^*1118*^*CS*

Aripiprazole is an off-target inhibitor of CI, and flies maintained on a diet supplemented with this drug exhibit damaged mitochondria [6]. Therefore, we asked whether their defects in spermatogenesis are due to compromised mitochondrial function. Mitochondrial health and function can be determined by analysing their membrane potential (Δψm). We measured Δψm via tetramethylrhodamine methyl ester (TMRM) in the spermatogonial region of live dissected testes. Flies kept on an aripiprazole containing diet presented a decrease in the Δψm, indicating that this drug impairs mitochondrial function in the testes (Fig. 2a, b). CI is also the major site of ROS production [29]. Excessive levels of ROS can damage cells, eventually leading to death [30]. We thus measured mitochondrial ROS (mtROS) in the spermatogonial region of the testes via MitoSOX, a mtROS indicator. We found that flies kept on a diet containing aripiprazole presented significant increases in ROS levels in their testes (Fig. 2c, d). We conclude that an aripiprazole-supplemented diet compromises mitochondrial function in the spermatogonial region.

We next examined whether functional compromise of mitochondria by aripiprazole altered their structure. Mitoballs are structures formed from aggregated mitochondria that emerge in the primary spermatocytes after mitosis [21].We used *BamGal4* to express mitochondria-targeted GFP to label the mitoball, and measured their size. We found that an aripiprazole-containing diet led to an increase in mitoball diameter at the late spermatocyte stage (Supplementary Fig. 2a-c). We conclude that an aripiprazole-supplemented diet disrupts the mitoball structure in late spermatocytes.

### An aripiprazole-supplemented diet decreases the number of spermatogonial cells

**Fig. 3:**
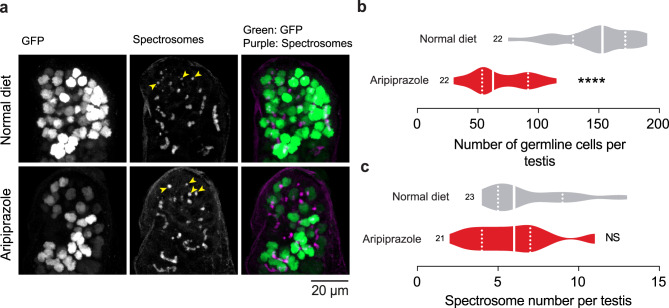
Flies supplemented with aripiprazole have a reduced number of spermatogonia. (**a-c**) Male flies kept on food supplemented with aripiprazole for 14 days have a reduced number of germline cells (spermatogonia). Representative confocal images (**a**) of *Drosophila* testes showing spermatogonia detected using a nuclear GFP reporter (green) and spectrosomes using immunofluorescence against Hts-IBI (magenta; yellow arrowheads). Quantification (**b**) of the number of GFP-positive nuclei (asterisks; unpaired t-test; n indicates the number of analysed testes), and quantification (**c**) of the number of spectrosomes per testis (NS, not significant; unpaired t-test; n indicates the number of analysed testes). Genotypes: (**a-c**) *w;UAS nuclear GFP/+;nosGal4/+*

Mitochondrial dysfunction can cause cell death (reviewed in [31]). Because flies kept on an aripiprazole-supplemented diet had compromised mitochondrial function in their spermatogonial region, we next measured the number of spermatogonial cells in this region of the testes. We used *nanos*Gal4 to express a nuclear localised GFP to label germline stem cells (GSCs), gonioblasts (GBs) and spermatogonial cells at the 2-, 4- and 8-cell stages (see Fig. 1a for details). We found that there was a significant reduction in the number of GFP-positive cells in the testes of flies kept on an aripiprazole-containing diet (Fig. 3a, b).

The cells within each spermatogonial cyst are interconnected by a large, branched organelle called the fusome. These structures persist in the male testes throughout mitotic and meiotic cell divisions. Fusomes coordinate the death of spermatogonial stages after DNA damage [20] and allow the movement of proteins between the cells within a spermatogonial cyst [32]. The fusomes in GSCs and GBs have a punctate appearance, and an increased number of punctate fusomes, which are called spectrosomes, is normally associated with either over proliferation of GSCs or defects in their differentiation [33, 34] (reviewed in [35]). The cellular levels of ROS regulate stemness in many cell types (reviewed in [36]). High ROS in testes results in the loss of germ cells via increased differentiation [37]. We observed increased ROS levels in the spermatogonial region of the testes in flies supplemented with an aripiprazole-containing diet, therefore we assayed both their differentiation and proliferation. We used an antibody against Hts-1B1, the *Drosophila* homologue of adducin, a protein found in the fusomes/spectrosomes [38].We counted the number of punctae (spectrosomes) as a readout for the differentiation/proliferation defects, and found no significant differences (Fig. 3a, c).

**Fig. 4:**
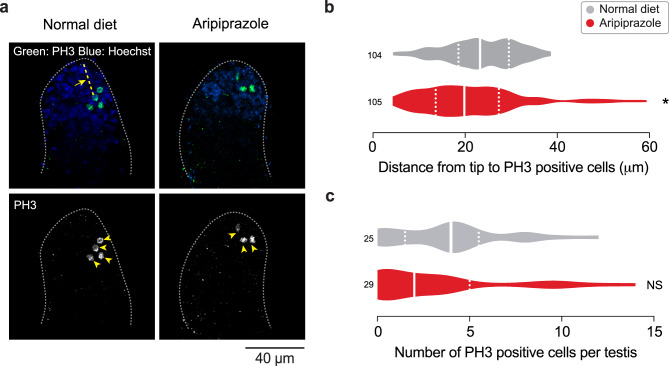
Aripiprazole supplementation does not alter cell proliferation. (**a-c**) The number of mitotic cells remain unchanged while their position is closer to the testes tip in male flies kept on food supplemented with aripiprazole for 14 days. Representative confocal images (**a**) of *Drosophila* testes, showing cells positive for phospho-histone H3 (PH3) (green; yellow arrowheads), and cell nuclei (blue) Quantification (**b**) of the distance from the tip of the testis to each PH3 positive cell (yellow arrow pointing to yellow dotted line) (asterisks; Mann‒Whitney test; n indicates the number of PH3 positive cells representing 25–29 testes). Quantification (**c**) of the number of PH3 positive cells (NS, not significant; Mann‒Whitney test; n indicates the number of testes). The analysis was performed on 14-day-old males. Genotypes: (**a-c**) *w;UAS nuclear GFP/+;BamGal4/+*

Next, to test whether the decrease in spermatogonial numbers is due to changes in the proliferation of GSCs, we measured their proliferation using an antibody against phospho-histone H3 (PH3), a mitotic proliferation marker. We found that PH3-positive cells were restricted to the anterior spermatogonial region, close to the tip of the testis, in flies kept on both an aripiprazole-supplemented and normal diet. We measured the distance from the tip of the testes to the PH3 positive cells and found a significant reduction in testes from flies kept on an aripiprazole-supplemented diet (Fig. 4a, b). We next counted these cells but found no significant alterations in the number of proliferating cells in the testes of flies kept on an aripiprazole-supplemented diet (Fig. 4a,c). We conclude that feeding flies with aripiprazole decreased the number of spermatogonial cells without altering either their proliferation or differentiation.

### An aripiprazole-supplemented diet increases lysosomal markers in spermatogonial cysts

**Fig. 5:**
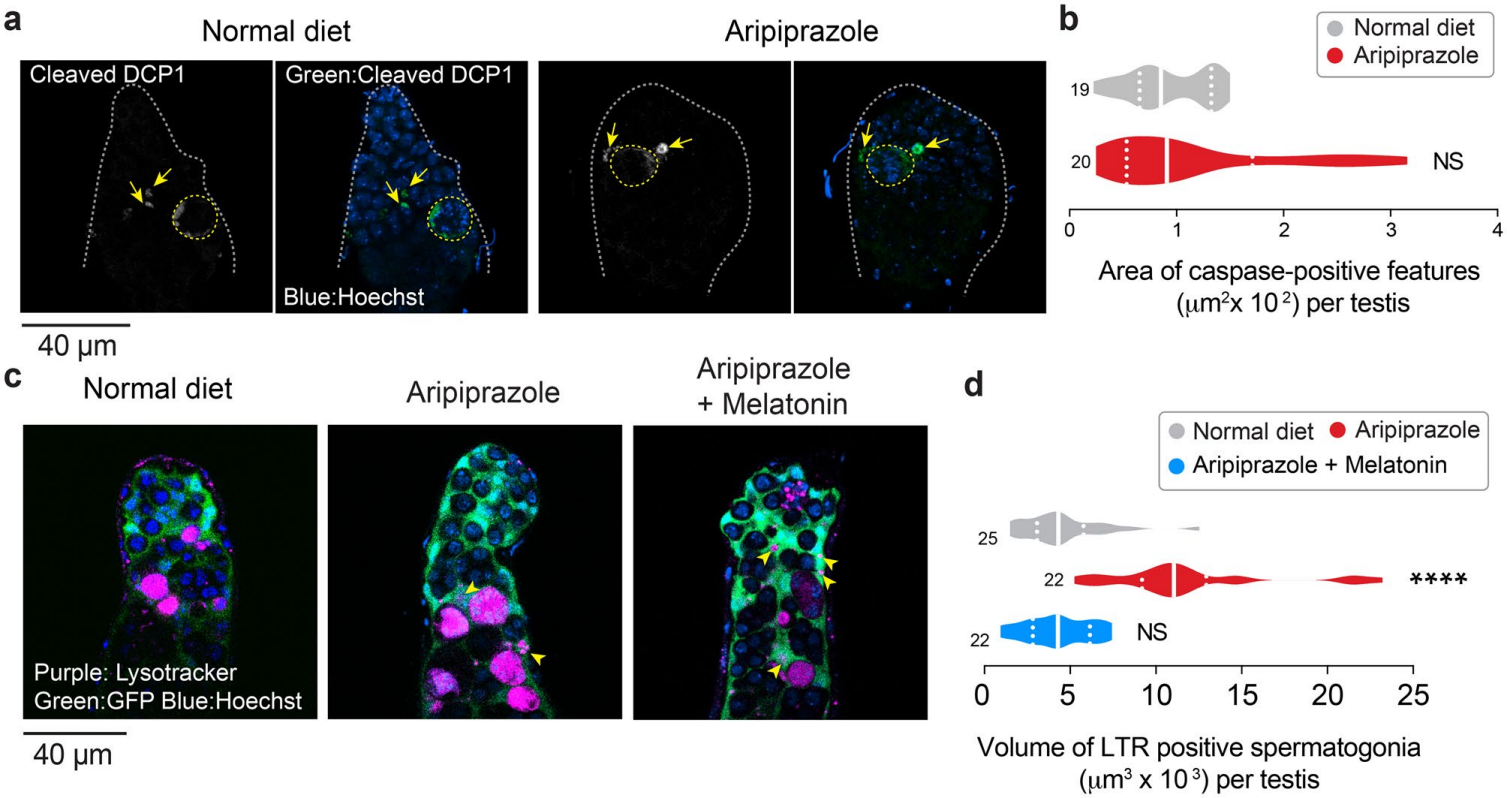
Antioxidant supplementation reduces the expression of markers of lysosomal degradation in the spermatogonia of flies kept on diet supplemented with aripiprazole. (**a, b**) Active Dcp-1, an effector caspase, is detected in the cyst cells around the spermatogonia (yellow dotted outline) and other cellular debris (yellow arrows) in both flies on normal diet and flies kept on an aripiprazole-containing diet. Representative confocal images (**a**) of active Dcp-1-positive structures detected using immunofluorescence against cleaved Dcp-1 in the spermatogonial region of the fly testis (area surrounded by the dotted grey line) and quantified (**b**) in flies kept on a normal diet and a diet supplemented with aripiprazole (NS, not significant; unpaired t-test; n indicates the number of testes). (**c, d**) The increase in the volume of LysoTracker red (LTR)-positive spermatogonia in files kept on an aripiprazole-containing diet was reduced by antioxidant supplementation. Representative confocal images (**c**) of live testes expressing GFP in cyst cells stained with LysoTracker Red, with some LysoTracker internalising within cyst cells (yellow arrowheads) and total volume of LysoTracker staining quantified in (**d**). Male flies were kept on a normal diet, an aripiprazole-containing diet or a diet supplemented with both aripiprazole and melatonin (asterisks; Kruskal-Wallis multiple comparisons with post-hoc Dunn’s test; n indicates the number of testes). The analysis was performed on 14-day-old males. Genotypes: (**a, b**) *w;BamGal4/+* and (**c, d**) c587*Gal4;UAS GFP/+*

Flies kept on an aripiprazole-supplemented diet presented a reduced number of spermatogonial cells but no significant changes in the proliferation or differentiation of GSCs. One of the two cyst cells surrounding a developing spermatogonia can undergo apoptosis, a form of programmed cell death, under stressful conditions such as starvation. This process is followed by the engulfment of the spermatogonial cyst by the surviving cyst cell [27]. Because high levels of ROS can cause cellular damage leading to apoptosis [30], we next measured the levels of active Dcp-1, a caspase involved in the apoptotic program in *Drosophila* cyst cells. We used an antibody against active (cleaved) Dcp-1 to stain apoptotic cells. We detected Dcp-1 positivity in the cyst cells surrounding the spermatogonia (Fig. 5a, dotted yellow outline and Supplementary Fig 3a, b, arrows) and other smaller structures possibly corresponding to cellular debris (Fig. 5a, arrows). However, we found no significant differences in the total area of testes stained for this apoptotic marker between flies kept on an aripiprazole-supplemented diet and normal diet (Fig. 5a, b). We conclude that the cyst cells in the flies kept on an aripiprazole-containing diet do not show increased cell death.

Spermatogonial cells contain an alternative cell death pathway that involves both mitochondrial and lysosomal components [39]. This alternative cell death pathway is observed in conditions such as stress-induced starvation [26] and irradiation-induced DNA damage [20]. We therefore stained the testes of flies kept on an aripiprazole-supplemented diet with a live lysosomal marker. We used *BamGal4* to label the spermatogonial cells with GFP and found increased Lysotracker Red staining in these cells, in the testes of males kept on aripiprazole containing diet (Supplementary Fig. 3c, d, arrows). We next used a marker of germline cyst cells (*c587-Gal4*) to label the cyst cells surrounding the spermatogonia with GFP. We next dissected these testes and stained them with LysoTracker Red dye. We detected increased labelling of spermatogonial cysts for LysoTracker Red as well as an increase in the volume of spermatogonial cysts positive for this lysosomal marker (Fig. 5c, d) in testes from flies kept on an aripiprazole-supplemented diet. We also observed an increase in small fragments of LysoTracker-positive structures within the cyst cells (Fig. 5c, arrowheads). This increase in LysoTracker staining was blocked when melatonin, a mitochondria-targeted antioxidant (reviewed in [40]), was supplemented along with aripiprazole in the fly diet (Fig. 5c, d). We conclude that a diet containing aripiprazole reduces the number of spermatogonia by their loss via lysosomal degradation, and this effect is suppressed by an antioxidant supplement.

### An aripiprazole-containing diet increases JNK activity in cyst cells

**Fig. 6:**
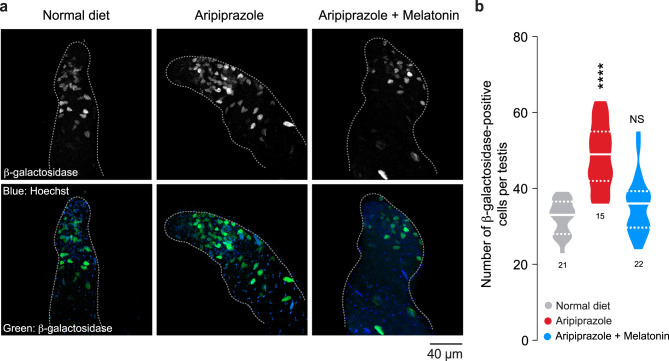
An antioxidant reduces the increased JNK signalling in the cyst cells of flies kept on a diet containing aripiprazole. (**a, b**) Aripiprazole activates the JNK pathway in cyst cells, as detected using the *puckered-lacZ* reporter, and this activation can be reduced by supplementation with antioxidants. Representative confocal images (**a**) of *Drosophila* testes with JNK activity detected in the spermatogonial region (area surrounded by the dotted grey line), visualised using a *β*-galactosidase antibody and quantified in (**b**). Male flies were kept on a normal diet, an aripiprazole-containing diet or a diet supplemented with both aripiprazole and melatonin (significance; one-way ANOVA with Tukey’s multiple comparisons test; n indicates the number of testes). The analysis was performed on 14-day-old males. Genotypes: (**a, b**) *pucE*^*69*^*/TM3, Sb*^*1*^

The JNK pathway is an important evolutionarily conserved stress‒response pathway that can be activated by ROS (reviewed in [41]). The JNK pathway in *Drosophila* is regulated by Basket (Bsk), which, upon activation, can activate the transcription of target genes whose activity aims to reduce cellular stress. JNK (Bsk) also activates the transcription of *puckered (puc),* which negatively regulates the pathway (reviewed in [42]). As the JNK pathway plays an important role in countering high ROS levels in many tissues, we measured the activation of this pathway in the testes of flies kept on a diet containing aripiprazole. The *puckered-lacZ* enhancer trap line [43] can be used as a tool to monitor JNK activity by immunostaining tissues with an anti-β-galactosidase antibody. We found flies kept on an aripiprazole-supplemented diet had an increased number of β-galactosidase-positive cyst cells in the spermatogonial region of the testis (Fig. 6a, b and Supplementary Fig. 4b). To test whether this increase in JNK activity was dependent on ROS, we kept flies on a diet containing aripiprazole-supplemented with the antioxidants melatonin or α-lipoic acid. We found that an antioxidant-supplemented diet led to a decreased number of β-galactosidase-positive cyst cells in the spermatogonia (Fig. 6a, b and Supplementary Fig. 4a, c).

**Fig. 7:**
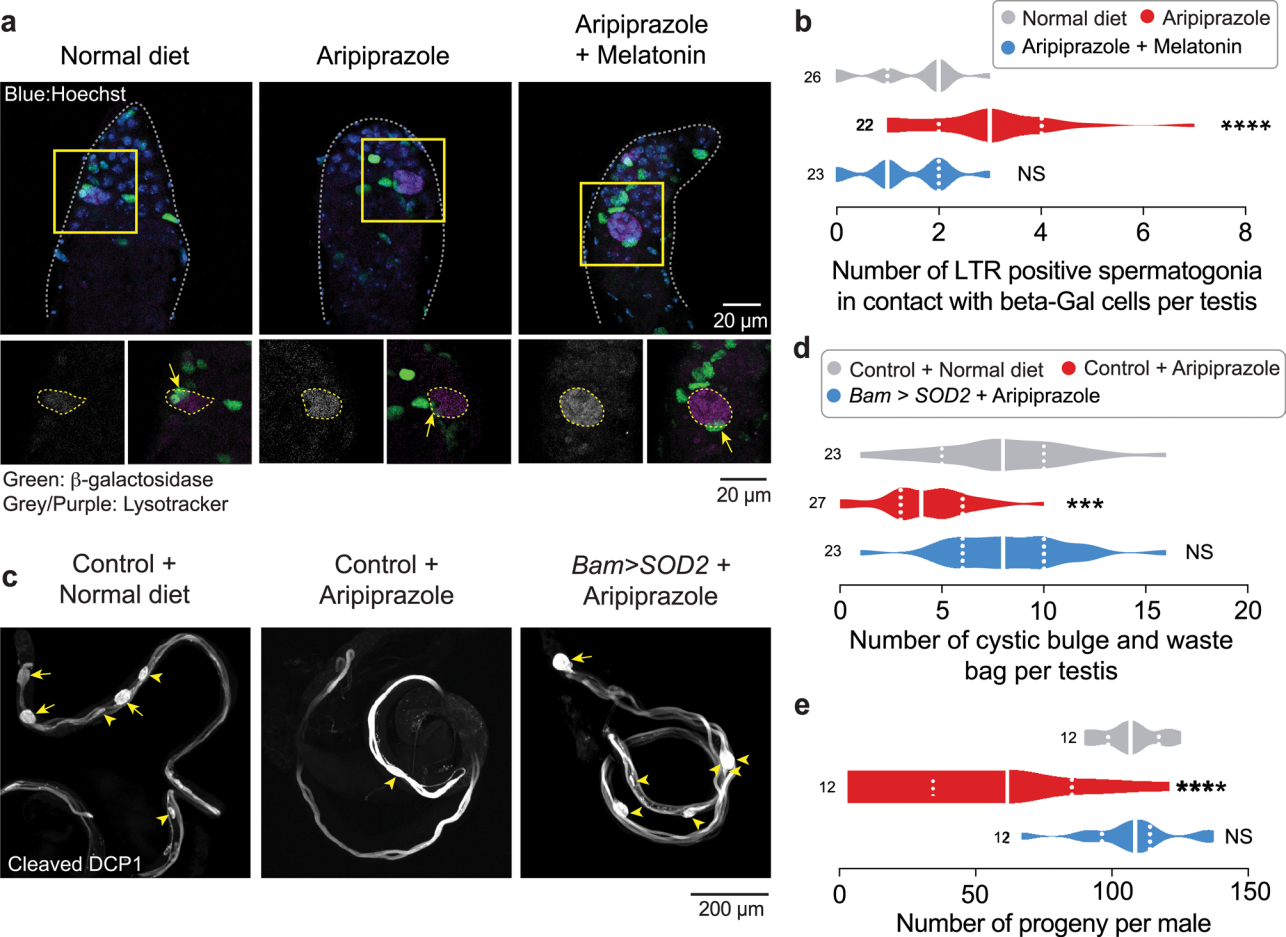
Supplementation with aripiprazole increases the association of LysoTracker-positive spermatogonia with JNK-positive cyst cells and the expression of mitochondrial superoxide dismutase in spermatogonia reduces aripiprazole associated fertility defects. (**a, b**) Flies kept on an aripiprazole-supplemented diet show an increase in the number of LysoTracker Red-positive spermatogonia associated with JNK-positive cyst cells, which is reduced by dietary supplementation with antioxidants. Representative confocal images (**a**) of *Drosophila* testes stained with LysoTracker Red (LTR) and immunofluorescence against a *β*-galactosidase antibody to detect JNK activity in the spermatogonial region (area surrounded by a dotted grey line). The bottom panels show high-magnification images of the yellow boxed regions. Quantification (**b**) of the number of LTR-positive spermatogonia (yellow dotted line; bottom panels) associated with lacZ-positive cyst cells (yellow arrows; bottom panels) (asterisks; one-way ANOVA with Tukey’s multiple comparisons test; n indicates the number of testes). The analysis was performed on 14-day-old males. (**c, d**) Spermatid individualisation defects observed in flies kept on an aripiprazole-supplemented diet were reduced by increasing the expression of SOD2 in spermatogonial cells. Representative confocal images (**c**) showing cystic bulges (yellow arrowheads) and waste bags (yellow arrows) detected using immunofluorescence against active Dcp-1 and quantification (**d**) of the total number of cystic bulges plus waste bags (significance; one-way ANOVA with Tukey’s multiple comparisons test; n indicates the number of testes). (**e**) The reduction in offspring from male flies kept on an aripiprazole-supplemented diet was reversed by the increased expression of SOD2 in spermatogonia (significance; one-way ANOVA with Tukey’s multiple comparisons test; n indicates the number males). The analysis results from crosses between males kept on an aripiprazole-supplemented or normal diet for 14 days and 2–3-day-old virgin *w*^*1118*^ females maintained on standard food. Genotypes: (**a-b**) pucE^*69*^*/TM3, Sb*^*1*^ (**c-e**) Control: *w;BamGal4/+*, and Bam > SOD2: *w;UAS SOD2/+;BamGal4/+*.

Under normal physiological conditions, cyst cells in testes can engulf (phagocytose) spermatogonial cells via a JNK-dependent pathway when the spermatogonia show intense LysoTracker staining [44]. As we observed an increased volume of LysoTracker-stained spermatogonia in the testes of flies kept on a diet supplemented with aripiprazole (Fig. 5c, d), we next tested whether the cyst cells with high JNK activity were associated with the LysoTracker-positive spermatogonia. We stained *puckered lacZ* testes with LysoTracker Red and assessed JNK activity using antibody staining against LacZ. We counted the number of LysoTracker Red positive spermatogonia (Fig. 7a, dotted yellow line) that are in contact with a β-galactosidase-positive cyst cell (Fig. 7a, arrows). We found an increase in the number of LysoTracker Red positive spermatogonia in contact with β-galactosidase-positive cyst cells in flies supplemented with an aripiprazole containing diet (Fig. 7a, b). To test whether this association was dependent on ROS, we kept the flies on a diet containing aripiprazole supplemented with melatonin. We found that in this case, there was a decrease in the number of LysoTracker-positive spermatogonia in contact with a β-galactosidase-positive cyst cell (Fig. 7a, b).

We conclude that an increase in ROS in the testes caused by aripiprazole activates the JNK pathway in cyst cells and there is an increased association of JNK-positive cyst cells with LysoTracker-positive spermatogonia.

### Aripiprazole-mediated spermatid individualisation and fertility defects are due to increased mitochondrial ROS

We found that aripiprazole increases the lysosomal degradation of spermatogonial cells in the testes. Next, we checked whether these flies presented any defects in the downstream stages of spermatid individualisation. Besides acting in the execution phase of apoptosis in the cyst cells, caspases play other important roles in *Drosophila* spermatid individualisation. F-actin investment cones strip away excess cytoplasm and cellular material in the form of cystic bulges (CBs). These CBs move along the length of the spermatids and are deposited at the tail end in a waste bag (WB), where they are degraded by active caspases (see Fig. 1a for details). *Drosophila* contain between 8 and 11 CBs and WBs structures per testes [45] and these can be detected by staining against active Dcp-1. Changes in the number of these structures, reflect defects in the individualisation process of spermatogenesis. The testes of flies kept on a diet containing aripiprazole were stained for active Dcp-1 and we found that they had a reduced number of cystic bulges (CBs) and waste bags (WBs) (Fig. 7c, d). Next, we determined whether the loss of Dcp-1-positive CB and WB was due to the increase in ROS in the spermatogonial cells caused by the oral administration of aripiprazole. We used the *BamGal4* driver to express mitochondrial superoxide dismutase 2 (SOD2) and suppress ROS in spermatogonial cells. SOD2 is a mitochondrial enzyme that dismutates highly reactive forms of ROS into less harmful compounds such as oxygen and hydrogen peroxide (reviewed in [46]. We found that the SOD2-mediated suppression of ROS in spermatogonial cells resulted in an increase in the number of CBs and WBs in flies kept on a diet supplemented with aripiprazole (Fig. 7c, d).

To determine whether the decreased fertility in flies kept on an aripiprazole-supplemented diet (Fig. 1g) is mediated by increased mtROS in the spermatogonia, we maintained both control male flies and male flies expressing SOD2 in spermatogonial cells on a diet containing aripiprazole for 14 days. We then set-up crosses between single male flies maintained on aripiprazole-supplemented diet and five female virgins kept on standard diet. We compared the number of progenies generated to that obtained from crosses with single males maintained on a normal diet. We found that the expression of SOD2 in the spermatogonial cells of male flies maintained on an aripiprazole-supplemented diet suppressed their fertility defects (Fig. 7e). We conclude that aripiprazole influences the proper spermatid individualisation process and male fertility through excess mtROS in the spermatocytes.

### Silencing a subunit of mitochondrial complex I in spermatogonial cells results in lysosomal degradation

**Fig. 8:**
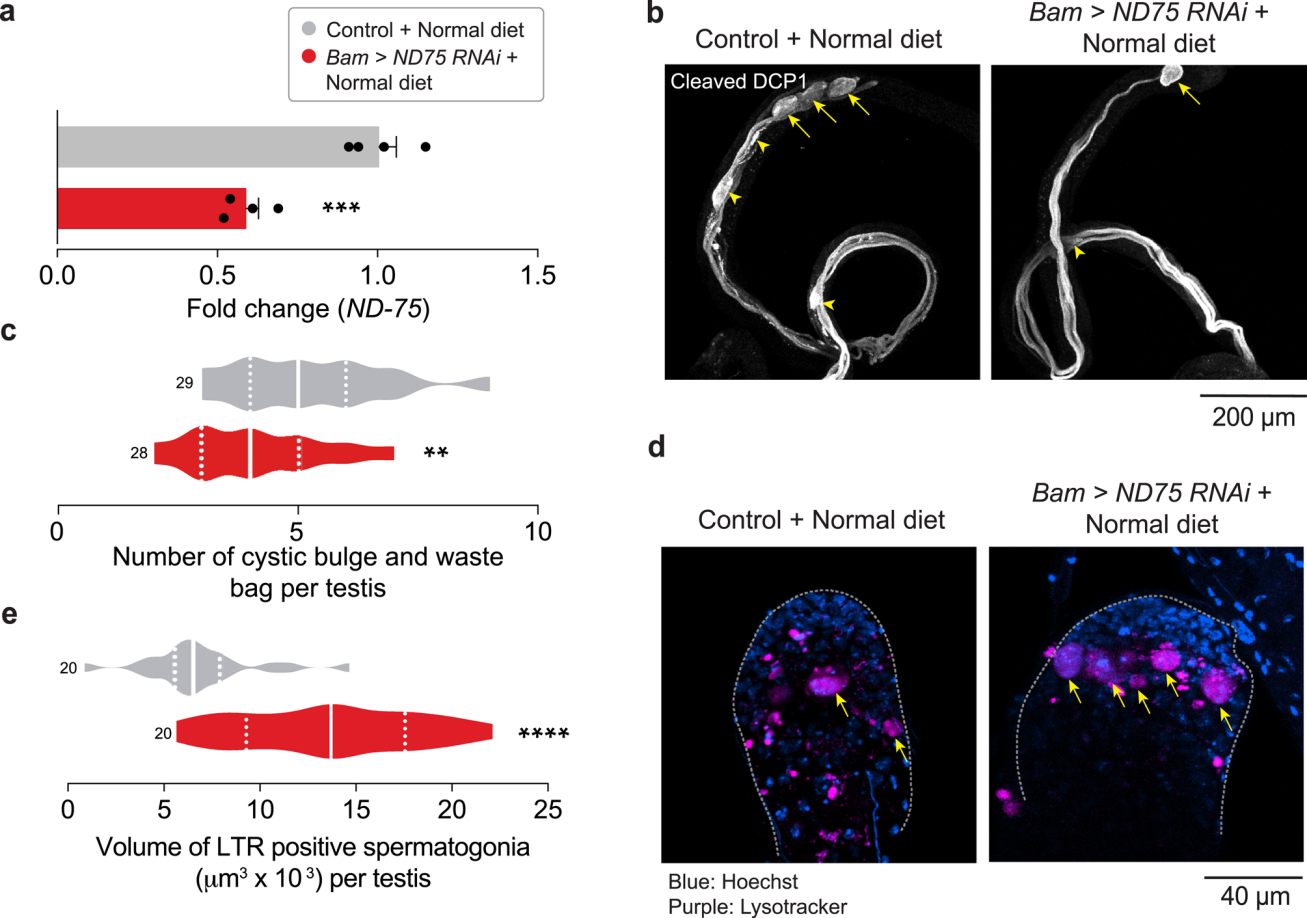
RNAi knockdown of a subunit of mitochondrial complex I in spermatogonia causes defects in spermatid individualisation and increases lysosomal markers. (**a**) Quantification of RNA upon knockdown of *ND-75* in spermatocytes using *BamGal4* (mean ± s.e.m.; asterisks; unpaired t-test; representing 80 males per condition). (**b, c**) RNAi knockdown of the mitochondrial complex I subunit ND-75 in spermatogonia reduces the number of caspase-positive cystic bulges and waste bags. Representative confocal images (**b**) of cystic bulges (yellow arrowheads) and waste bags (yellow arrows) detected using immunofluorescence against active dcp-1 and quantification (**c**) of the total number of cystic bulges and waste bags (asterisks; unpaired t-test; n indicates the number of testes). (**d, e**) *ND-75* knockdown in spermatogonia increases the volume of LysoTracker Red-positive spermatogonia in *drosophila* testes. Representative confocal images (**d**) of LysoTracker Red (LTR) staining (present in the area surrounded by the dotted grey line) showing LysoTracker-positive spermatogonial cysts (yellow arrows). Quantification (**e**) of the volume of LysoTracker Red-positive spermatogonia (asterisks; unpaired t-test; n indicates the number of testes). All analysis was performed on 14-day-old males. Genotypes: (**a-e**) control: *w;BamGal4/+* and Bam > ND-75RNAi: *w;BamGal4/UAS ND-75RNAi*

The findings thus far support the notion that aripiprazole causes toxicity to the reproductive system of male flies through off-target inhibition of CI. To corroborate these findings, we investigated whether CI deficiency has effects on the reproductive system of fruit flies analogous to the ingestion of aripiprazole. Earlier studies have shown that the knockdown of CI protein subunits results in reduced fertility in flies, reduction in the number of CBs and WBs [47] and increased germ cell differentiation in the testes [48]. However, whether CI deficiency in spermatogonial cells increases the loss of these cells via lysosomal degradation is unknown. We first measured the levels of active Dcp-1 in testes where ND-75, a CI component, was silenced by RNAi in spermatogonial cells using the *BamGal4* driver. We found that *ND-75* knockdown in spermatogonia decreased its transcript levels (Fig. 8a) and it resulted in a decrease in the number of caspase-positive CBs and WBs (Fig. 8b, c).

Next, we assessed whether the enhanced lysosomal degradation of spermatogonial cells observed in flies kept on an aripiprazole-supplemented diet also occurred after a direct compromise of CI. We stained the testes of flies expressing *ND-75 *RNAi in spermatogonia with a live lysosomal marker. We found that, compared with the control, suppression of this CI subunit significantly increased the volume of LysoTracker-positive spermatogonia (Fig. 8d, e).

We therefore conclude that the knockdown of mitochondrial CI in spermatogonia causes the loss of spermatogonial cells via lysosomal degradation and a reduction in the number of individualised spermatids at later stages.

## Discussion

Using *Drosophila* as a model, we showed that aripiprazole compromises fertility in male flies. Mitochondrial damage caused by the ingestion of aripiprazole results in increased ROS. Excessive ROS levels result in increased spermatogonial cell death via lysosomal degradation. This subsequently compromises spermatid individualisation and decreases fertility.

Aripiprazole is a partial agonist of mammalian dopamine D_2_ receptors (D_2_Rs). The *Drosophila* genome encodes two D_1_ and one D_2_-like receptor (reviewed in [49], but their expression level in the testes is low [50]. The low expression levels of D_2_-like receptors in *Drosophila* testes makes it unlikely that the activation of dopamine signalling by aripiprazole is an important contributing factor to damage to the male reproductive system.

Dopamine is an important neurotransmitter that regulates courting behaviour in both male and female flies, and inhibiting dopamine synthesis in female flies results in reduced mating receptivity [51]. In contrast, decreased dopamine signalling has no effect on male mating behaviour [51]. A recent study found that dopaminergic signalling in the blood brain barrier cells of adult *Drosophila* males influences their courtship behaviour [28]. Dopamine levels in the PPL2ab neuronal cluster of *Drosophila* males regulate mating drive [52]. We did not find significant changes in transient male courtship behaviour of flies kept on an aripiprazole-containing diet. However, we did not perform a longitudinal analysis of courtship to completely rule-out an effect of aripiprazole on long-term mating behaviour.

The Or47b olfactory receptor neurons detect fly pheromones and are essential for male mating success [53]. The juvenile hormone is an age- related regulator of reproductive maturity in males and acts by increasing the sensitivity of the Or47b neurons to sex pheromones [54]. The sensitivity of Or47b neurons is also regulated by population density [55]. The activity of Or47b neurons in 7-day-old males is increased if they are housed in groups of 10 or more [54, 55]. In our experiments, we used male flies that were older than 11 days, housed in groups of 15 to 20 individuals. It is therefore unlikely that population density accounts for Or47b-dependent alterations of fertility measured in our experimental settings. Dopamine does not directly modulate the activity of Or47b neurons. However, dopamine affects olfactory signal-associated memory formation [56]. We did not perform learning tests in male flies in our study. However, we restored the fertility of males kept on an aripiprazole-containing diet by reducing the ROS levels in spermatogonial cells. We therefore reason that the effect of aripiprazole on fertility is linked directly to the compromise of spermatogonial cells with little or no contribution from a compromise of the nervous system. In mammals, in addition to dopamine receptors, aripiprazole also binds to several serotonin receptors (5-HT1A, 5-HT2B, and 5-HT7) with relatively high affinity [57]. Orthologues of these receptors are present in the *Drosophila* genome; however, their expression in the male testes is low [50]. In *Drosophila,* 5-HT7 receptor-mediated signalling also influences male courtship behaviour [58], but it is unknown whether it affects male fertility.

Reactive oxygen species (ROS) are generated primarily by mitochondria (reviewed in [59]), and ROS-mediated signalling plays an important role in spermatogenesis in both mammals and in *Drosophila* [37]. High levels of ROS can be detrimental to cellular function and differentiation and are associated with 30–80% of cases of infertility in men (reviewed in [60]). Exposure to xenobiotics can increase mitochondrial ROS production by blocking electron transport, leading to an increase in the reduction capacity of upstream carriers, or by directly transferring electrons from carriers to molecular oxygen (reviewed in [61]). We have shown that aripiprazole inhibits complex I through its ubiquinone binding channel [6]. Inhibition of this site is associated with increased ROS production [62]. We found that flies kept on an aripiprazole-containing diet had a reduced mitochondrial membrane potential and increased mtROS in the spermatogonial region of the testes (Fig. 2a–d).

Excessive levels of ROS are buffered via the antioxidant capacity of cells, that involves enzymes such as superoxide dismutase (SOD), catalase (Cat) and glutathione peroxidase (GPx) (reviewed in [63]). Elevated ROS levels can overwhelm cellular antioxidant defences and cause oxidative stress. Oxidative stress results in a loss of stemness in mammalian testes and triggers the apoptosis of spermatogonial cells (reviewed in [64]). Similarly, increased ROS in *Drosophila* testes results in a reduction in the stem cell population via increased differentiation of germ cells [48]. However, whether excess mtROS in *Drosophila* testes influences germ cell death (GCD) is unknown.

GCD is a physiological phenomenon in which a large percentage of spermatogonial cells in both *Drosophila* and mammals spontaneously undergo programmed cell death [39, 65, 66]. Unlike in mammalian testes, GCD in flies occurs independently of effector caspases but requires both lysosomes and mitochondria [39]. GCD is increased in the presence of stressors such as starvation and DNA damage [20, 26]. Spermatogonial cells develop within cysts surrounded by two cyst cells that support their development (Fig. 1a). During prolonged protein starvation, caspase-dependent apoptosis of a cyst cell triggers GCD of the spermatogonia [26, 27]. This results in their acidification and death, which is followed by their engulfment by the surviving cyst cell [27]. In our study, we did not detect any alterations in effector caspase activation in the cyst cells between males kept on aripiprazole-supplemented and normal diets (Fig. 5a and b). We therefore reason that aripiprazole-induced spermatogonial cyst acidification and death can occur independently of cyst cell apoptosis.

The inhibition of CI by xenobiotics or the suppression of ND-75, a mitochondrial complex I protein subunit, can induce ROS and disrupt cellular homeostasis in *Drosophila* testes [37]. We found that the knockdown of *ND-75* in spermatogonia increased GCD with increased spermatogonial acidification and lysosomal degradation (Fig. 8d, e). This finding is consistent with the concept that aripiprazole exerts its off-target effects via the inhibition of CI. Omi/HtrA2, mitochondrial serine protease that regulates apoptosis [67] was previously identified as being required for GCD [39], spermatid individualisation and fertility [68]. The mitochondrial permeability transition pore (mPTP) controls ROS levels and acts as a regulator of mitochondrial homeostasis (reviewed in [69]). High ROS levels influence the duration of pore opening. This can lead to local mitochondrial damage that can spread further to compromise cell viability (reviewed in [69]). The prolonged opening of the mPTP caused by ROS may result in the release of proapoptotic factors, including Omi/HtrA2, from mitochondria in spermatogonial cells, initiating the process of GCD.

The c-Jun N-terminal kinase (JNK) pathway is an important regulator of the cellular homeostasis of *Drosophila* testes (reviewed in [70]) and can be activated by ROS (reviewed in [71]). The function of JNK can be pro- or antiapoptotic and is influenced by factors such as cell type, death stimulus, activation duration, and other signalling pathways (reviewed in [72]). We detected a robust increase in the expression of markers of JNK activation in the cyst cells of flies kept on an aripiprazole-supplemented diet (Fig. 6a and b). However, we did not detect an increase in active Dcp-1, an effector caspase, in these cells (Fig. 5a and b). Therefore, the increase in JNK activation in these cells does not increase caspase-dependent cell death. In rat hepatoma cells, prior exposure to oxidative stress dampens JNK phosphorylation and reduces cell death upon prolonged exposure to aripiprazole [73]. Historical exposure to stress may therefore alter cellular responses to aripiprazole toxicity.

We also did not observe any significant increase in the level of PH3, a mitotic marker, in the spermatogonial region of the testes. Therefore, the increased number of cyst cells positive for JNK in flies kept on a diet supplemented with aripiprazole is unlikely to be due to an increase in cell division in response to stress. We found a reduction in the distance of the PH3 cells from the tip of the testes, supporting a loss of spermatogonial cells in files kept on an aripiprazole-supplemented diet.

JNK activation in multiple cell types regulates their ability to engulf neighbouring cells by phagocytosis. In *Drosophila*, JNK activation is necessary in glial cells for the phagocytic clearance of damaged neurons [74] and in cyst cells in testes for the phagocytosis of spermatogonial cells by neighbouring healthy cyst cells [44]. We found that an increased number of LysoTracker-positive spermatogonia were associated with JNK-positive cyst cells in the testes of flies kept on a diet supplemented with aripiprazole (Fig. 7a and b). Our results suggest that JNK pathway activation in cyst cells may facilitate their engulfment and elimination of damaged spermatogonia.

Extracellular ROS released from dying cells can activate JNK signalling in the neighbouring cells via TNF receptor signalling [75]. In a similar manner, ROS released from damaged spermatogonia can activate TNF receptor signalling, which acts upstream of JNK signalling in cyst cells. Alternatively, intrinsic ROS in cyst cells could also activate JNK via dASK1 (apoptosis signal regulating kinase-1) (reviewed in [70]). The mechanism of JNK activation in cyst cells in the presence of excess mtROS and whether such activation is linked to TNF-receptor signalling, will require further investigation.

We showed that higher levels of mitochondrial ROS in the spermatogonial cells result in their loss via the GCD pathway. This reduces the number of spermatids, impacting fertility. The defects observed at later spermatid stages (Fig. 1b–e and Fig. 7c–d) may occur in spermatogonia that escaped GCD, contributing to the fertility defects. Aripiprazole is ingested orally and is present in the whole organism. In this study, for practical reasons, we did not measure aripiprazole levels in the testes of the flies. However, we reason that once the drug enters the fly haemolymph, it should diffuse into all tissues. All developmental stages of spermatogenesis are present in adult *Drosophila* testes at any given time point. It is possible that aripiprazole may also compromise mitochondrial function by directly interfering in the late developmental stages of spermatids. This could be tested by using a driver like *Rbp4Gal4* [76], to knockdown mitochondrial CI in spermatocytes and spermatids to better characterise the consequences of the loss of CI for late stages of spermatogenesis.

The use of antipsychotic drugs is increasing both in the UK [77] and globally (reviewed in [78]). Antipsychotics with reduced side-effect profiles, such as aripiprazole, are valuable psychiatric tools. These antipsychotics are also being increasingly used in children, adolescents [79] and younger men [77]. Our findings suggest that the use of antipsychotics in younger males might have off-target effects on their reproductive system. Although preclinical safety data suggests that aripiprazole has no reproductive or developmental toxicity, there are no comprehensive studies in this area. While patients taking aripiprazole report gastrointestinal discomfort, damage to spermatogonia and sperm is more likely to go unnoticed. Unlike most first and second generation antipsychotic medications, aripiprazole tends to decrease the levels of prolactin hormone in young and adolescent males [80], the long term consequences of which are unknown. Reduced prolactin levels are associated with reduced testosterone levels [81], reduced sperm quality and compromised fertility in adults [82].

Patients treated with antipsychotics, including aripiprazole show a reduction in sperm concentration and motility [83]. In mice, administration of aripiprazole leads to morphological damage in the developing spermatids and loss of spermatogonial cells [8, 9]. Whether these effects are due to reduced prolactin levels alone or a combined effect of both hormone and mitochondrial dysfunction in spermatogonial cells and developing spermatids, is unknown. It is also unknown if these sperm effects are reversed following termination of the treatment. While termination of treatment may not be practical for a number of patients, our findings suggest that antioxidant supplements could be useful to mitigate sperm defects. Antioxidant supplements can protect the male reproductive system from off-target ROS damage caused by drugs such as aripiprazole, but caution is needed when these compounds are combined with antipsychotics. More studies are required to understand the benefits and limitations of antioxidant supplements when taken alongside these drugs.

## Methods

### *Drosophila* strains

Fly stocks were maintained on standard cornmeal agar media. The flies were grown and analysed under a light/dark cycle of 12 h/12 h at 25 °C. We used the *w*^*1118*^*CS, Canton-S* strain as a control. This is the standard control strain used in the lab and was generated by backcrossing *Canton-S* to *w*^*1118*^ for 5–7 generations [84].This strain has wild type *white* gene, in an otherwise *w*^*1118*^ background. The following lines were obtained from the Bloomington stock centre: JNK reporter line, *pucE*^69^*/TM3, Sb*^1^ (#98329); cyst cell driver, *PGawBC587* (#67747); *yv;UAS ND-75 RNAi* (#27739); *w;UAS SOD2* (#24494); *w;UAS mito-GFP* (#95270); *UAS nuclear GFP;* and *w;nosGAl4* (Hansong Ma, University of Birmingham); *w;UAS GFP* (John Roote, Fly facility, University of Cambridge); and *w;Bam Gal4* (Helen White-Cooper, University of Cardiff).

### Drug treatments

Aripiprazole (Abcam, cat.no. ab120764), dissolved in DMSO, was incorporated into liquified standard cornmeal agar food at a final concentration of 1 mM. After thorough mixing the liquid was poured into fresh empty fly food vials, allowed to cool and used as ‘drug supplemented food’. We incorporated aripiprazole into the fly food at a concentration established in previous studies [6, 14]. We used chronic feeding for 11 to 14 days, as we have previously shown that there are no major physiological changes associated with this feeding protocol [6] and the detected levels of the drug in flies are comparable to its human blood plasma levels [14].

Flies kept on a diet supplemented with aripiprazole were compared to flies kept on a normal diet supplemented with 1% (v/v) DMSO. For experiments involving antioxidants, melatonin (Merck, M5250) was dissolved in DMSO (0.43 mM) and α-lipoic acid (Sigma-Aldrich, 437692) was dissolved in DMSO (2.15 mM). Diet containing both aripiprazole and melatonin or α-lipoic acid was prepared by combining both chemicals in liquified cornmeal agar food at final concentrations of 1 mM aripiprazole and 0.43 mM melatonin or 1 mM aripiprazole and 2.15 mM α-lipoic acid in 1% (v/v) DMSO. We used a concentration of melatonin or α-lipoic acid reported in a previous study [85]. Freshly hatched unmated males were kept on drug-supplemented diet in groups of 15–20 males per vial for the specified duration (11–14 days) to avoid complications due to changes in germ cell homeostasis after mating [86] or low food intake [26]. The flies were transferred to a fresh drug supplemented food (same batch of origin), every 3rd day until required for experiments.

### Light and electron microscopy

Morphological assessment of mitochondria in spermatids was performed via phase contrast microscopy with a 40x objective on a Zeiss Axioplan2 imaging system. To obtain phase contrast images, the testes were dissected in PBS at room temperature and placed in a drop of PBS on a glass slide. A small nick was made in the anterior part of the testes, and a coverslip was placed gently over the tissue. Excess buffer was blotted from the edge of the coverslip, and the liquid displacement helped spread out the different cell types from the dissected testes. The sample was imaged immediately.

For TEM, testes were dissected and fixed overnight at 4 °C in 0.1 M sodium cacodylate buffer (pH 7.4) containing 2% paraformaldehyde, 2.5% glutaraldehyde and 0.1% Tween 20. The samples were subsequently fixed for 1 h at room temperature in a solution containing 1% osmium tetroxide and 1% potassium ferrocyanide. Post fixation, the samples were stained en bloc with 5% aqueous uranyl acetate overnight at room temperature. The samples were dehydrated via a series of ethanol washes and embedded in Taab epoxy resin (Taab Laboratories Equipment Ltd., Aldermaston, UK). semi thin sections were stained with toluidine blue, and areas of the sections were selected for ultramicrotomy. Ultrathin sections were stained with lead citrate and imaged using a TemCam XF416 digital camera and EM Menu software (TVIPS, Gilching, Germany) with a Jeol 1400 electron microscope (Jeol UK Ltd., Welwyn Garden City, UK).

### Fertility assay

Newly hatched males were kept on a diet supplemented with aripiprazole or DMSO for 14 days. Each adult male fly was placed in a vial with 5 *w*^*1118*^ virgin females (3–5 days old) at 25 °C. After 24 hours, the male was removed, and the mated females were placed individually in separate vials and allowed to lay eggs for either two consecutive days, in data reported in Fig. 1g, or three consecutive days, in data reported in Fig. 7e, after which the females were removed. The vials with eggs were allowed to develop and hatch into adults. All adults were removed, and the number of empty pupal cases was recorded as the number of live offspring. Only vials with pupae were included in the quantification across all conditions. The average number of offspring per male was measured by dividing the total number of offspring from each male by the number of females from which they were obtained.

### Courtship assay

Single-pair courtship assays were performed using *w*^*1118*^*CS* flies adapting established methods. [87]. Newly hatched males were maintained on a diet supplemented with aripiprazole or a normal diet for an 11-day period. Virgin females, 2- to 3-day-old, kept on a standard diet were used for setting up the courtship assay. Mating pairs were housed in single wells of 96-well, white, flat-bottomed plates (Greiner Bio-one CELLSTAR, Greiner 655083), each well containing a single drop of yeast paste. Each pair underwent a 5-minute acclimation process. Mating pairs were subsequently recorded using a mobile phone camera secured to a tripod. We recorded movies for a duration of 25 to 30 minutes. To minimise the impact of circadian rhythms on mating behaviour, experiments were conducted at a consistent circadian time, specifically from 08:30 hrs to 10:00 hrs (GMT) over a two-day period.

We manually analysed movies for each of the recorded matings. We measured both the display and duration of courtship behaviour. We defined the courtship index as the fraction of time males spent displaying courtship behavior. Latency to court is the measured time until the male begins the courtship display.

### Body weight measurements

Body weight measurements were performed as previously described [88]. In short, five groups of seven adult males were anesthetised and weighed in a plastic tray. The weights were obtained at four different time points (3, 7, 14 and 25 days-old). The body weight, in mg, was normalised to the number of flies measured.

### Microscopy-based assessment of mitochondrial structure, function, ROS and LysoTracker staining in live testes

Measurements of the Δψm in *Drosophila* testes were performed in a manner similar to that used for *Drosophila* midgut tissue staining described previously [89]. Briefly, the testes were dissected in PBS and incubated for 40 min at room temperature with 40 nM TMRM (Thermo Scientific, cat.no. T668) in loading buffer (10 mM HEPES pH 7.35, 156 mM NaCl, 3 mM KCl, 2 mM MgSO_4_, 1.25 mM KH_2_PO_4_, 2 mM CaCl_2_, and 10 mM glucose), and the dye was present during the experiment. In these experiments, TMRM was used in redistribution mode to assess the Δψm; therefore, a reduction in TMRM fluorescence represents mitochondrial depolarisation. Confocal images were obtained using a Zeiss LSM 980 confocal microscope equipped with a 20x air objective. The fluorescence was quantified by exciting TMRM with a 561 nm laser and measuring above 580 nm.

Measurements of MitoSOX Red (Thermo Scientific, cat.no. M36008) were performed similarly to TMRM. The testes were dissected in sterile Schneider’s media and incubated with 30 nM MitoSOX Red for 30 minutes in the dark. At the end of the incubation period, the testes were washed once in Schneiders and mounted in fresh media to be imaged immediately. The fluorescence was quantified by exciting MitoSOX with a 514 nm laser and measuring it above 580 nm. Confocal images were obtained using a Zeiss LSM 980 confocal microscope equipped with a 20x air objective.

For measurements of LysoTracker Red (Thermo Scientific, cat.no. L7528), the testes were dissected in HBSS (containing calcium and magnesium, phenol red free) and incubated with 50 nM LysoTracker Red for 10 minutes at room temperature in the dark. At the end of the incubation period, the testes were washed once with 1X HBSS, mounted in the same buffer and imaged immediately. Images were acquired by exciting LysoTracker Red using a 565 nm laser and measuring above 580 nm.

For measurements of mitochondrial morphology, the testes from the relevant genotypes and treatments were dissected in HBSS (containing calcium and magnesium, phenol red free), and incubated with Hoechst 33342 (1:500, Invitrogen, cat.no. H3570) for 5 minutes and imaged immediately.

Confocal images were obtained using a Zeiss LSM 980 confocal microscope equipped with a 40x water or a 20x air objective. The laser illumination intensity was maintained at a minimum (0.1- 0.5% of the laser output) to avoid phototoxicity, and the pin hole was set such that an optical slice of 0.5 or 1 μm was obtained.

### Immunofluorescence and confocal microscopy

For immunostaining, testes were dissected in ice-cold PBS, fixed in 4% PFA for 30 minutes and blocked overnight in 5% bovine serum albumin with PBS/0.5% Triton X-100. The samples were then incubated with the following primary antibodies: anti-GFP (Abcam, cat. no. ab13970, 1:1000), anti-PH3 (CST, cat.no. 9713S and 9701S, 1:200 each), anti-Hts-IBI (DSHB, AB 528070, 1:50), anti-cleaved *Drosophila* Dcp-1 (CST, cat.no. 9678, 1:100), anti-β-galactose (Promega, cat.no. Z378A, 1:500) at 4 °C overnight and then with the relevant secondary antibodies (Alexa Fluor™ 488-conjugated F(ab’)2 fragment of goat anti-rabbit IgG (H + L) (1:500, Invitrogen, cat. no. A11070), Alexa Fluor™ 488-conjugated goat anti-mouse IgG (H + L) (1:500, Invitrogen, cat.no. A11029), Alexa Fluor 488-conjugated goat anti-chicken IgY (H + L) (1:200, Abcam, cat.no. ab150169) and Hoechst 33342 (1:500, Invitrogen, cat.no. H3570) at room temperature for 2 hours or at 4 °C overnight. The samples were washed in PBS/0.5% Triton X-100, mounted in Vectashield (Vector Laboratories cat.no. H-1000) and stored at 4 °C, and images were acquired using a Zeiss LSM 980 confocal system.

### Digital image processing and image analysis

Fluorescence images were acquired as uncompressed bitmapped digital data (TIFF and CZI formats) and processed via Fiji, which employs established scientific imaging workflows.

To obtain the fluorescence intensity (from TMRM and MitoSOX red staining), the spermatogonial region of the testis was selected (area marked with dotted yellow lines in Fig. 2a and c). Within this area, 5 regions of interest (ROIs) of 10 μm^2^ were selected and fluorescence intensity measured. The mean fluorescence intensity per testis was obtained from these, for each group.

To obtain the number of spermatogonia labelled with *nosGal4* driving *UAS nuclear GFP*, the total volume of the GFP signal was first measured using the 3D objects counter in Fiji (a size threshold of 1 μm^3^ was set to remove background staining). The average volume of a nucleus was obtained by measuring the diameter of 15 random nuclei from 3 different images per condition. The number of spermatogonia was then obtained by dividing the total volume of the GFP-positive signal by the volume of a single nucleus for each image.

The areas of Dcp-1-positive structures in the spermatogonial region were obtained from Z stacked images and manual thresholding (default type). The area measurement tool was used to obtain the area of the thresholded image.

The volume of LysoTracker Red staining was obtained using the 3D objects counter plugin in Fiji. A size threshold of 1 μm^3^ was set to exclude background structures in all of the samples. A volume measurement tool was used to obtain the volume of thresholded objects, and the values were summed to obtain the total volume of LysoTracker Red-positive structures for each sample.

The video recordings of the courtship assay were processed using kdenlive (version 25.04.0, KDE) and exported as MPEG-4 files at 10 times real time with embedded timestamps.

### RNA extraction and qRT-PCR

Total RNA was extracted from 20 freshly dissected pairs of male testes for each replicate using TRIzol (Ambion) and quantified by spectrophotometric analysis (Nanodrop, Thermo Scientific). We performed RT‒qPCR using a real-time cycler (Applied Biosystems 75000, Fast Real-Time PCR Systems) and the SensiFAST SYBR Lo-ROX One-Step Kit (Bioline). The fold-change in expression was calculated using the comparative Ct method [90]. For RT‒qPCR, we measured the coefficient of variance (CV) of the technical replicates, and any samples with a CV > 3% were excluded from statistical analysis. The gene-specific primers were ND-75 forward: 5′-ATCGGCGTGGAGATACCCA-3′ reverse: 5′-GTCAAATCCGAGTTGGTCTTGAT-3′

and RP49, forward: 5′-TGTCCTTCCAGCTTCAAGATGACCATC-3′; reverse: 5′-CTTGGGCTTGCGCCATTTGTG-3′) (Sigma‒Aldrich).

### Statistical analyses

No statistical methods were used to predetermine sample size. Statistical analyses were performed using GraphPad Prism (http://www.graphpad.com). The data are presented as the mean values, and the error bars indicate ± s.e.m. The number of biological replicates per experimental variable (n) is indicated in either the respective figure or the figure legend. No sample was excluded from the analysis unless otherwise stated. Blinding was not performed. Normality of the data for statistical analyses was assessed using the D’Agostino-Pearson test and appropriate statistical analyses were performed based on normality. Significance is indicated as: NS, not significant; *p* > 0.05; *p≤0.05; **p≤0.01; ***p≤0.001; and ****p≤0.0001. The statistical information for each individual figure and supplementary figure is provided in supplementary Table 1.

## Electronic supplementary material

Below is the link to the electronic supplementary material.

Supplementary Material 1

## Data Availability

All data generated or analysed during this study are included in this article and is available upon reasonable request to the corresponding authors, Amrita Mukherjee or L. Miguel Martins.

## Abbreviations

CI: mitochondrial respiratory complex I
Δψm: mitochondrial membrane potential
GCD: germ cell death
JNK: c-Jun N-terminal kinase
mtROS: mitochondrially derived reactive oxygen species
mPTP: mitochondrial permeability transition pore
ROS: reactive oxygen species
TMRM: tetramethylrhodamine methyl ester perchlorate
SOD2: superoxide dismutase 2 (mitochondrial)
LTR: LysoTracker Red
D_2_R: dopamine receptor D_2_
Dop2R: D_2_-like dopamine receptor
