# Supplementary material for: Oral intake of aripiprazole compromises male fertility in Drosophila
Source: Biol Direct. 2025 Nov 11;20:110. doi: 10.1186/s13062-025-00698-9 (PMC12607071; doi:10.1186/s13062-025-00698-9)
Supplement: Supplementary file 1 — Supplementary Material 1 [file 13062_2025_698_MOESM1_ESM.docx]

**Oral intake of aripiprazole compromises male fertility in *Drosophila***

*Supplementary Information*

## Supplementary Figures & Figure Legends


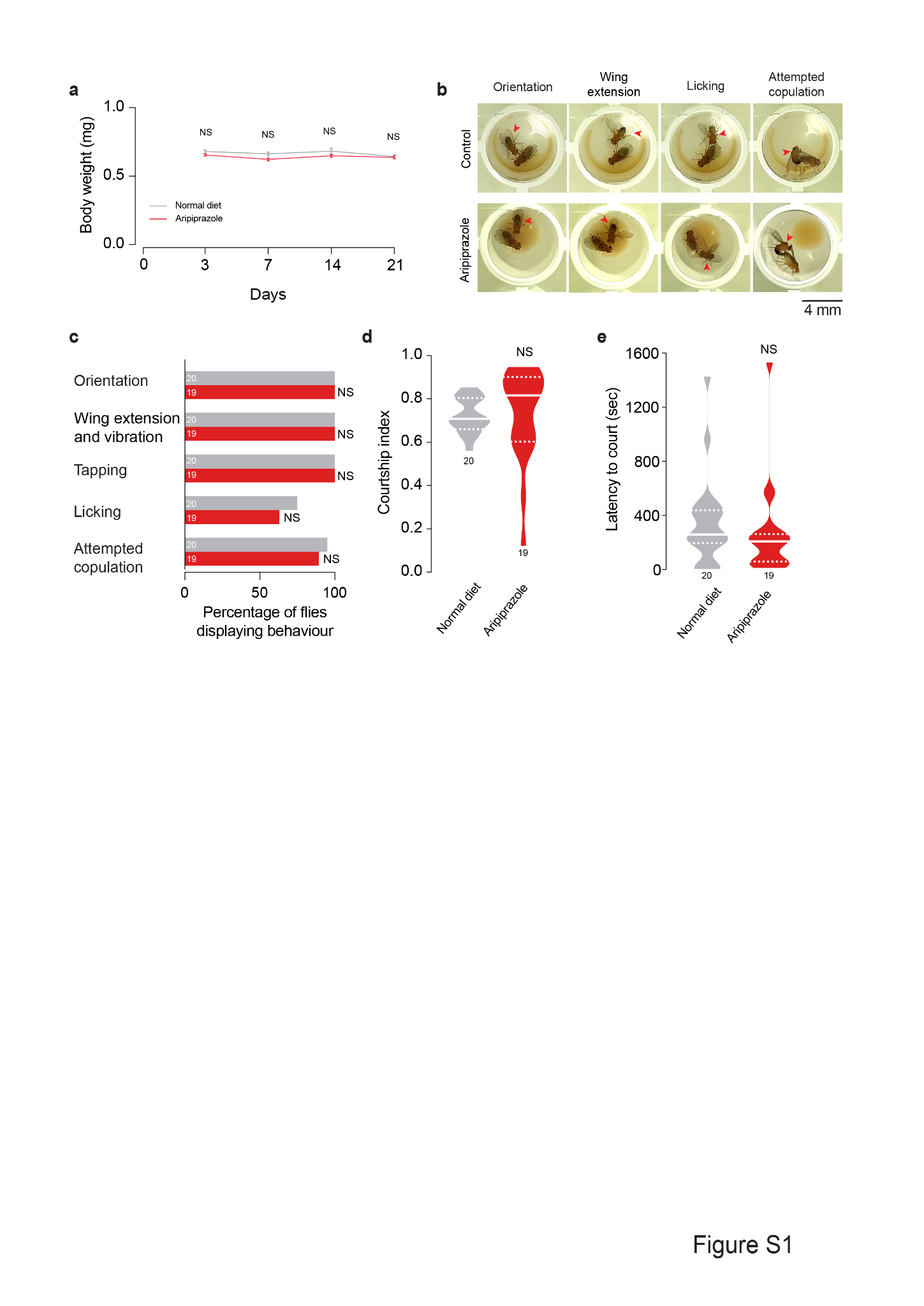


### Supplementary figure 1. Aripiprazole supplemented diet in males does not alter their body weight or disrupt male courtship.

(**a**) An aripiprazole-supplemented diet does not alter the body weight of male flies. (mean ± s.e.m.; NS, not significant; 2-way Anova with Šídák’s multiple comparisons test, 5 biological replicates each containing 7 males) (**b**-**e**) Male flies kept on an aripiprazole-supplemented diet for 11 days show normal courtship rituals. Representative images (**b**) of mating pairs, with males (red arrowheads) displaying courtship mating rituals and quantification (**c**) of the different male courtship behaviours (NS, not significant; Fisher’s exact test; n indicates the number of males in each analysis). (**d**) Aripiprazole-supplemented diet does not alter male courtship index, measured as the fraction of time each male spends displaying all of the mating ritual behaviours (NS, not significant; Mann‒Whitney test; n indicates the number of males). (**e**) Aripiprazole-supplemented diet does not alter the latency to court, measured as the time taken for a male to display first sign of courtship (NS, not significant; Mann‒Whitney test; n indicates the number of males). Genotypes: (**a**-**e**) *w^1118^CS.*


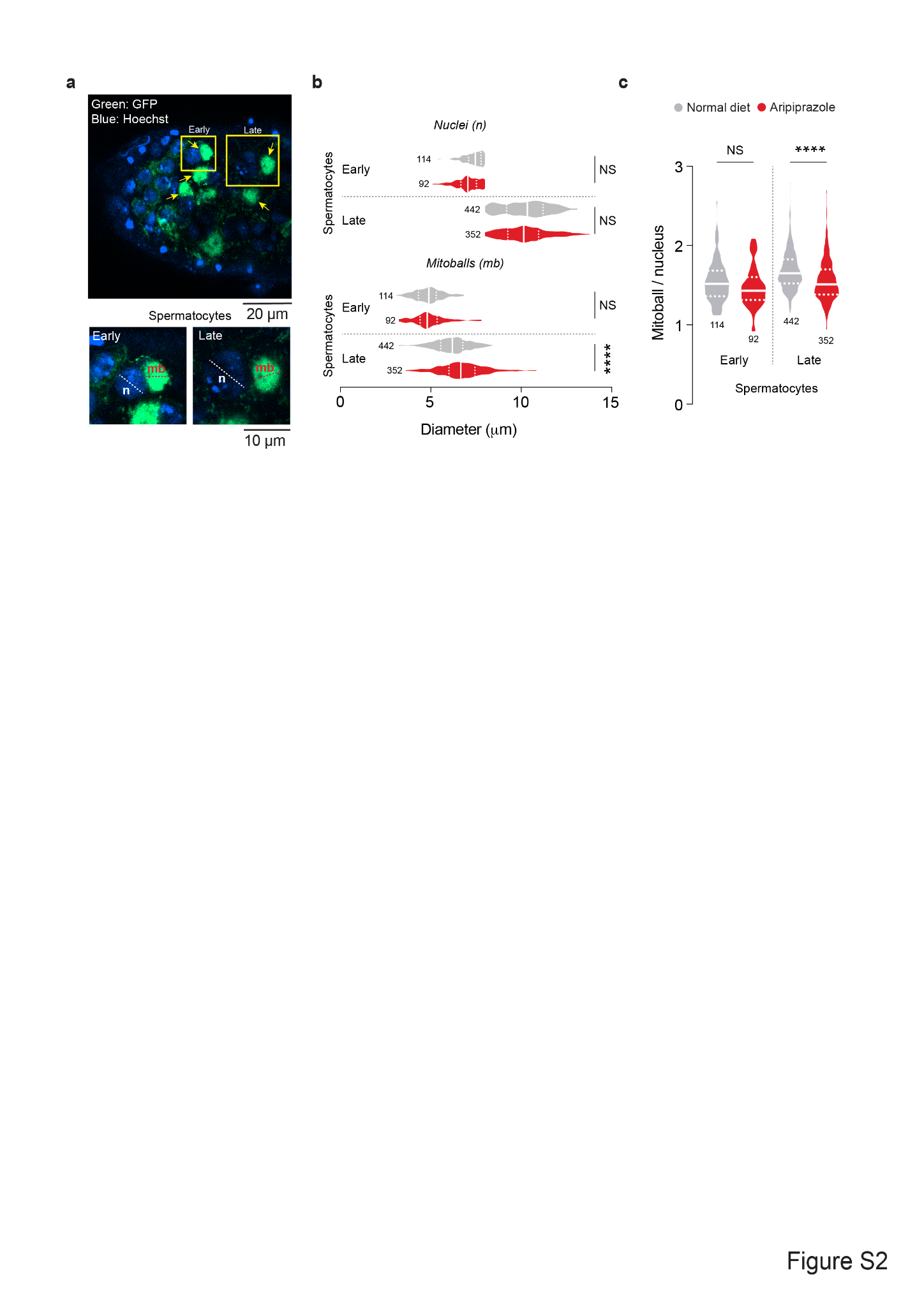


### Supplementary figure 2. An aripiprazole supplemented diet disrupts the mitoball structure in late spermatocytes.

(**a**-**c**) The mitoball structure is disrupted in late-stage spermatocytes in the testes of male kept on a aripiprazole-containing diet. Representative confocal images (**a**) showing mitochondria (green) forming the ‘mitoball’ structure (yellow arrows) in both early and late spermatocytes and cell nuclei (blue). The bottom panels show high-magnification images of the yellow boxed regions. The white dotted line across the nucleus (n) and the red dotted line across the mitoball (mb) represents the diameter measurements made. Nucleus diameter less than 8 μm was defined as ‘early’ spermatocyte and anything above as a ‘late’ spermatocyte (**b**-**c**) Aripiprazole-supplemented diet disrupts the diameter of mitoball in late spermatocytes (**b**) Quantification of the diameter of nuclei and mitoballs in early and late spermatocytes (asterisks; significance; Kruskal-Wallis multiple comparisons with post-hoc Dunn’s test; n indicates the number of spermatocytes representing 11 males per condition). (**c**) Quantification of the ratio of nuclei over mitoballs (asterisks; one-way ANOVA with Kruskal-Wallis multiple comparisons test; n indicates the number of spermatocytes representing 11 males per condition). The analysis was performed in 14-day-old males. Genotypes: (**a**-**c**) *w;;BamGal4/UAS mito-GFP.*


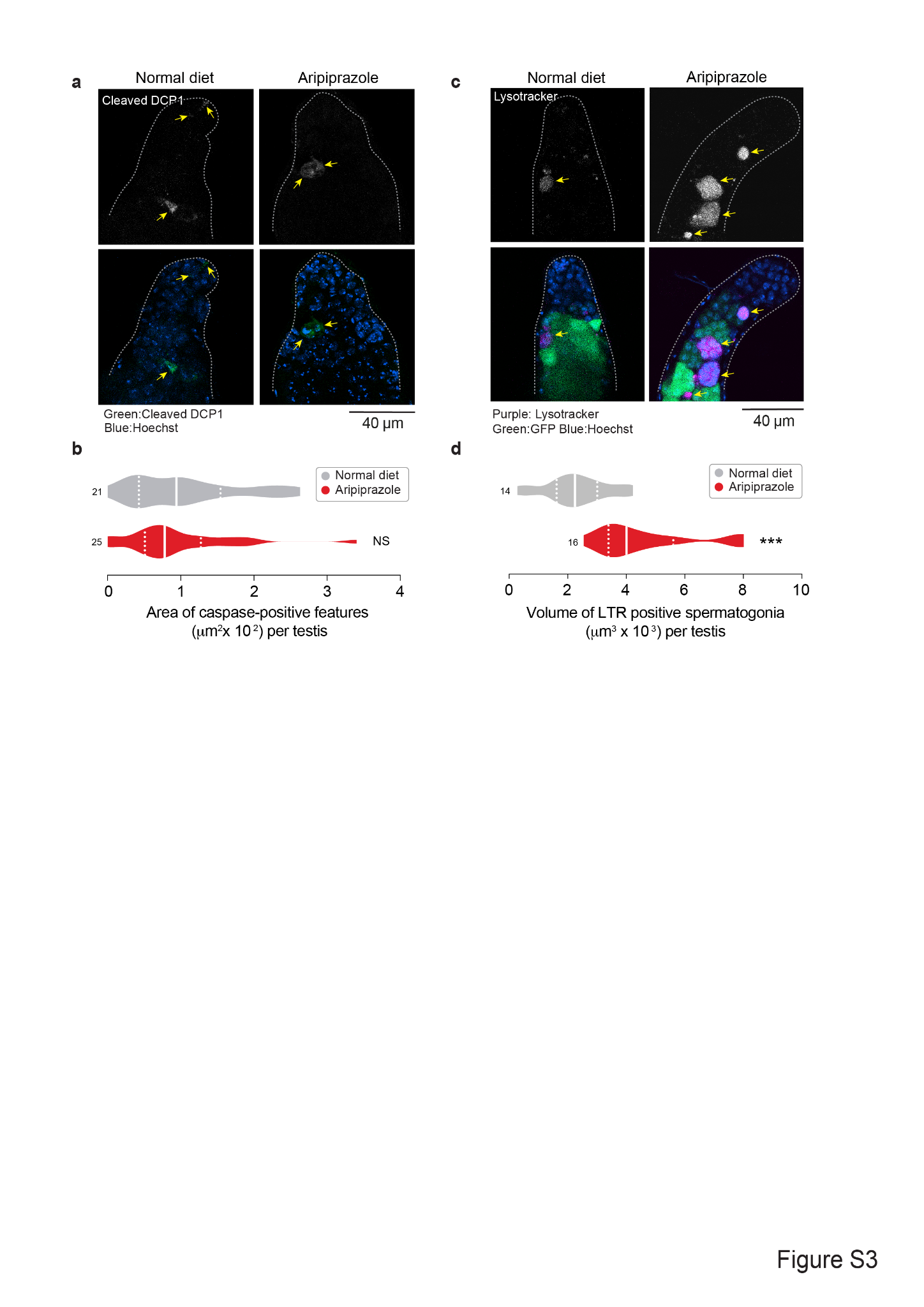


### Supplementary figure 3. Aripiprazole-supplemented diet does not alter active caspase levels in testes germarium but increases lysosomal markers in spermatogonia.

(**a**, **b**) Flies kept on a diet supplemented with aripiprazole do not show altered levels of the effector caspase Dcp-1 in the testes (yellow arrows). Representative confocal images (**a**) of active Dcp-1-positive structures detected using immunofluorescence against cleaved Dcp-1 in the spermatogonial region of the fly testis (area surrounded by the dotted grey line) and quantification (**b**) (NS, not significant; Mann‒Whitney test; n indicates the number of testes). (**c**, **d**) The volume of LysoTracker red (LTR)-positive spermatogonia in flies kept on aripiprazole-supplemented diet is increased. Representative confocal images (**c**) of live testes expressing GFP in spermatogonial cells and stained with LysoTracker Red, and quantification (**d**) of the volume of LTR positive spermatogonia (asterisks; unpaired t-test, n indicates the number of testes). This analysis was performed on 14-day-old males. Genotypes: (**a**, **b**) *w^1118^CS* and (**c**, **d**) *w;;BamGal4/UAS GFP.*


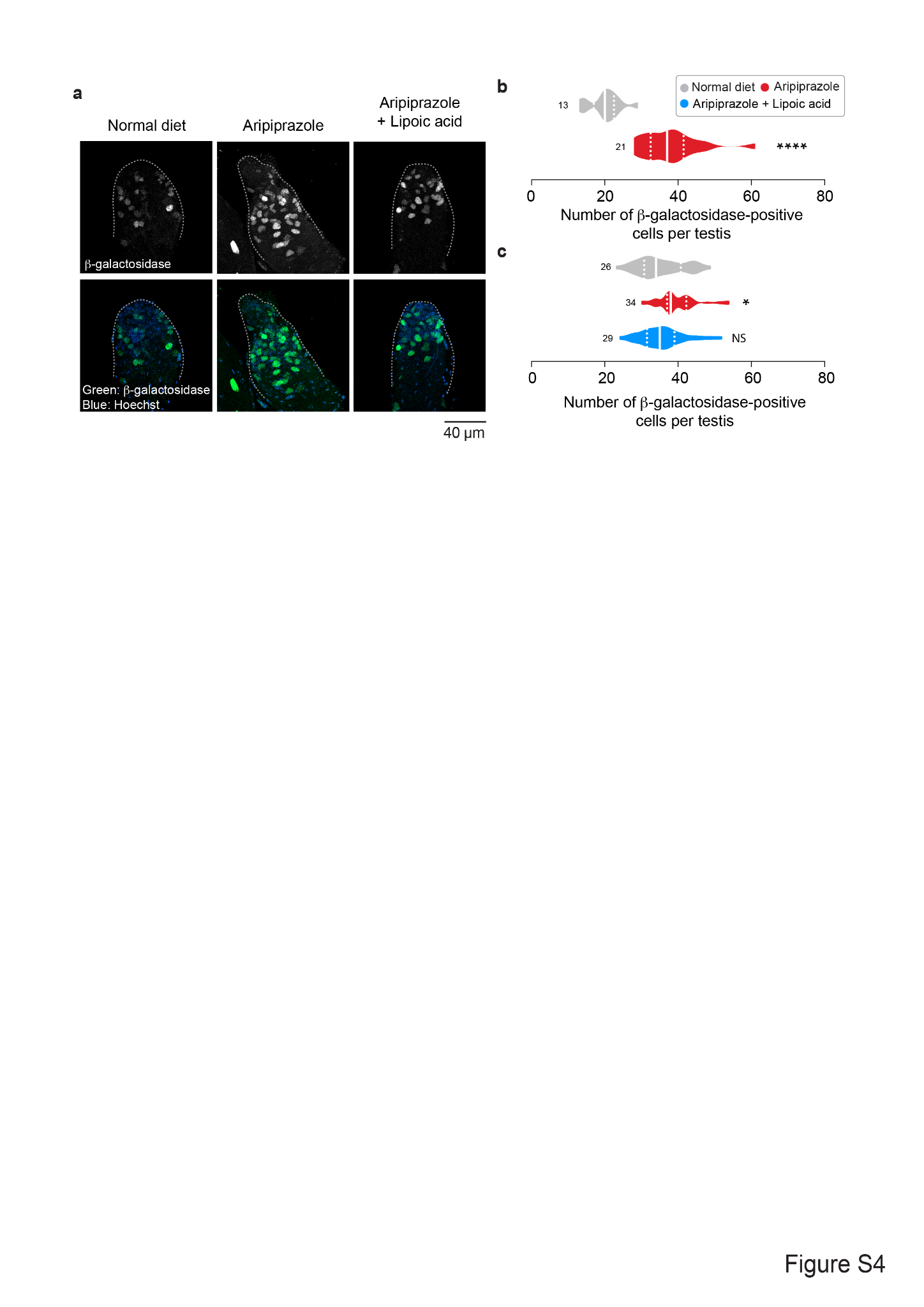


### Supplementary figure 4. Increased JNK signalling in the cyst cells of flies kept on aripiprazole supplemented diet is reduced by the antioxidant α-lipoic acid.

(**a**-**c**) Males kept on an aripiprazole-containing diet have increased activation of the JNK pathway in cyst cells, as detected using the *puckered-lacZ* reporter, and this activation can be reduced by supplementation with antioxidants. Representative confocal images (**a**) of *Drosophila* testes with JNK activity in the spermatogonial region (area surrounded by the dotted grey line), visualised using a *β*-galactosidase antibody. Quantification (**b**) of Jnk pathway activation, as measured by the number of *β*-galactosidase positive cells, in cyst cells in the testes of males kept on a diet supplemented with aripiprazole or a diet without aripiprazole (asterisks; Mann-Whitney test; n indicates the number of testes). Quantification (**c**) of Jnk pathway activation, as measured by the number of *β*-galactosidase positive cells, in cyst cells in the testes of males kept on a diet supplemented with aripiprazole, a diet supplemented with both aripiprazole and the antioxidant lipoic acid, or normal diet (asterisks; one-way ANOVA with Tukey’s multiple comparisons test; n indicates the number of testes). The analysis was performed on 14-day-old males. Genotypes: (**a**-**c**) *puc^E69^/TM3, Sb^1^*.

### Supplementary table 1.

Statistical information on the analysed data. Normality was assessed using the D'Agostino-Pearson test. con, control genotype; ari, aripiprazole-supplemented food; mel, melatonin-supplemented diet; LA, lipoic acid-containing diet; N/A, not applicable; N, nucleus; MB, mushroom body; ^1^, counted variables.

| **Figure number** | **Normal distribution?** | **Statistical test** | **P-value** | **significant** | **Standard deviation** | **Standard error of mean** | **n numbers (counted variables)^1^** | **Number of flies** |
| --- | --- | --- | --- | --- | --- | --- | --- | --- |
| 1c | N/A | Fisher's exact test, Two-sided | ≤0.0001 | yes | N/A | N/A | normal diet, 104; ari, 125  (onion stage spermatids) | normal diet, 5; ari, 4 |
| 1e | N/A | Fisher's exact test, Two-sided | ≤0.0001 | yes | N/A | N/A | normal diet, 117; ari,95 (post individualised spermatids) | normal diet, 2; ari, 2 |
| 1f | N/A | Fisher's exact test, Two-sided | ≥0.9999 | no | N/A | N/A | normal diet, 70; ari, 56 (post individualised spermatids) | normal diet, 2; ari, 2 |
| 1g | yes | unpaired t-test | 0.0249 | yes | normal diet, 11.62; ari, 9.722 | normal diet, 3.674; ari, 2.931 | normal diet, 10; ari, 11 (male flies) | normal diet, 10; ari, 11 |
| 2b 11 day TMRM | no | Mann-Whitney test | 0.0434 | yes | N/A | N/A | normal diet, 8; ari, 10 (testis) | normal diet, 4; ari, 5 |
| 2b 14 day TMRM | no | Mann-Whitney test | ≤0.0001 | yes | N/A | N/A | normal diet, 20; ari, 20 (testis) | normal diet, 10; ari, 10 |
| 2d 11 day MitoSOX | yes | unpaired t-test | 0.0405 | yes | normal, 353; ari, 683.1 | normal, 133.4; ari, 227.7 | normal diet, 7; ari, 9 (testis) | normal diet, 4; ari, 5 |
| 2d 14 day MitoSOX | no | Mann-Whitney test | ≤0.0001 | yes | N/A | N/A | normal diet, 24; ari, 22 (testis) | normal diet, 12; ari, 11 |
| 3b | yes | unpaired t-test | ≤0.0001 | yes | normal diet, 30.41; ari, 23.6 | normal diet, 6.484; ari, 5.031 | normal diet, 22; ari, 22 (testis) | normal diet, 11; ari, 11 |
| 3c | yes | unpaired t-test | 0.1685 | no | normal diet, 2.564; ari, 2.442 | normal diet, 0.5346; ari, 0.5328 | normal diet, 23; ari, 21 (testis) | normal diet, 12; ari, 11 |
| 4b | no | Mann-Whitney test | 0.0163 | yes | N/A | N/A | normal diet, 104; ari, 195 (PH3 positive cells) | normal diet, 13; ari, 15 |
| 4c | no | Mann-Whitney test | 0.2262 | no | N/A | N/A | normal diet, 25; ari, 29 (testis) | normal diet, 13; ari, 15 |
| 5b | yes | unpaired t-test | 0.2546 | no | normal diet, 38.81; ari, 86.35 | normal diet, 8.904; ari, 19.31 | normal diet, 19; ari, 20 (testis) | normal diet, 10, ari, 10 |
| 5d | no | Kruskal-Wallis test with Dunn's multiple comparison | normal diet vs ari, ≤0.000; normal diet vs ari + mel ≥0.999; ari vs ari + mel ≤0.0001 | normal diet vs ari, yes; normal diet vs ari + mel, no; ari vs ari + mel, yes | N/A | N/A | normal diet, 25; ari, 22; ari + mel, 22 (testis) | normal diet, 13; ari, 11; ari + mel, 11 |
| 6b | yes | Ordinary one-way ANOVA with Tukey's multiple comparison | normal diet vs ari, ≤0.0001; normal diet vs ari + mel, 0.3220; ari vs ari + mel, ≤0.0001 | normal diet vs ari, yes; normal diet vs ari + mel, no; ari vs. ari + mel, yes | normal diet, 4.501; ari, 8.593; ari + mel, 7.824 | normal diet, 0.9822; ari, 2.219; ari + mel, 1.668 | normal diet, 21; ari, 15; ari + mel, 22 (testis) | normal diet, 11; ari, 8; ari + mel, 11 |
| 7b | yes | Ordinary one-way ANOVA with Tukey's multiple comparison | normal diet vs ari, ≤0.0001; normal diet vs ari + mel, 0.9725; ari vs ari + mel, ≤0.0001 | normal diet vs ari, yes; normal diet vs ari + mel, no; ari Vs. ari + mel, yes | normal diet, 0.9047; ari, 1.430; ari + mel, 0.8913 | normal diet, 0.1774; ari, 0.3049; ari + mel, 0.1859 | normal diet, 26; ari, 22; ari + mel, 23 (testis) | normal diet, 13; ari, 11; ari + mel, 12 |
| 7d | yes | Ordinary one-way ANOVA with Tukey's multiple comparison | con + normal diet vs con + ari, ≤0.0001; con + normal diet vs Bam>SOD2 + ari, 0.9529; con + ari vs Bam>SOD2 + ari, ≤0.0001 | con + normal diet vs con + ari, yes; con + normal diet vs Bam>SOD2+ ari, no; con + ari vs Bam>SOD2 + ari, yes | con + normal diet, 3.483; con + ari, 2.301; Bam>SOD2 + ari, 3.169 | con + normal diet, 0.7263; con + ari, 0.4428; Bam>SOD2 + ari, 0.6607 | con + normal diet, 23; con + ari, 27; Bam>SOD2 + ari, 23 (testis) | con + normal diet, 12; con + ari, 14; Bam>SOD2 + ari, 12 |
| 7e | yes | Ordinary one-way ANOVA with Tukey's multiple comparison | con + normal diet vs con + ari, ≤0.0001; con + normal diet vs Bam>SOD2 + ari ≥0.9999; con + ari vs Bam>SOD2 + ari, ≤0.0001 | con + normal diet vs con + ari, yes; con + normal diet vs Bam>SOD2 + ari, no; con + ari vs Bam>SOD2 + ari, yes | con + normal diet, 10.83; con + ari, 33.49; Bam>SOD2 + ari, 18.66 | con + normal diet, 3.125; con + ari, 9.668; Bam>SOD2 + ari, 5.386 | con + normal diet, 12; con + ari, 12; Bam>SOD2 + ari, 12 (flies) | con + normal diet, 12; con + ari, 12; Bam>SOD2 + ari, 12 |
| 8a | n too small | unpaired t-test | 0.0008 | yes | con + normal diet, 0.1072; Bam>ND75RNAi + normal diet, 0.07703 | con + normal diet, 0.05362; Bam>ND75RNAi + normal diet, 0.03851 | con + normal diet, 4; Bam>ND75RNAi + normal diet, 4 (replicates each containing testis from 20 flies) | con + normal diet, 80; Bam>ND75RNAi + normal diet, 80 |
| 8c | yes | unpaired t-test | 0.0073 | yes | con + normal diet, 1.64; Bam>ND75RNAi + normal diet, 1.423 | con + normal diet, 0.3045; Bam>ND75RNAi + normal diet, 0.2689 | con + normal diet, 29; Bam>ND75RNAi + normal diet, 28 (testis) | con + normal diet, 15; Bam>ND75RNAi + normal diet, 14 |
| 8e | yes | unpaired t-test | ≤0.0001 | yes | con + normal diet, 2939; Bam>ND75RNAi + normal diet, 4699 | con + normal diet, 657.2; Bam>ND75RNAi + normal diet,1051 | con + normal diet, 20; Bam>ND75RNAi + normal diet, 20 (testis) | con + normal diet, 10; Bam>ND75RNAi + normal diet, 10 |
| S1a | n too small | 2 way ANOVA Šídák's multiple comparisons test | normal diet vs ari_day3, 0.3465; normal diet vs ari_day7,0.1614; normal diet vs ari_day14, 0.5771; normal diet Vs ari_day21, 0.9967 | normal diet vs ari_day3, no; normal diet vs ari_day7, no; normal diet vs ari_day14, no; normal diet Vs ari_day21, no | normal diet_day3, 0.026; normal diet_day7, 0.029; normal diet_day14, 0.047; normal diet_day21, 0.033; ari_day3, 0.016; ari_day7, 0.023; ari_day14, 0.023; ari_day 21, 0.023 | normal diet_day3, 0.011; normal diet_day7, 0.013; normal diet_day14, 0.021; normal diet_day21, 0.015; ari_day3, 0.007; ari_day7, 0.010; ari_day14, 0.010; ari_day21, 0.010 | normal diet, 5; ari, 5 (replicates each containing 7 flies) | normal diet_day3, 35; normal diet_day7, 35; normal diet_day14, 35; normal diet_day21, 35; ari_day3, 35; ari_day7, 35; ari_day 14, 35; ari_day21, 35 |
| S1c_licking behaviour | N/A | Fisher's exact test, Two-sided | 0.5006 | no | N/A | N/A | normal diet, 20; ari, 19 (flies) | normal diet, 20; ari, 19 |
| S1c_attempted mating behaviour | N/A | Fisher's exact test, Two-sided | 0.605 | no | N/A | N/A | normal diet, 20; ari, 19 (flies) | normal diet, 20; ari, 19 |
| S1d | no | Mann-Whitney test | 0.1249 | no | N/A | N/A | normal diet, 20; ari, 19 (flies) | normal diet, 20; ari, 19 |
| S1e | no | Mann-Whitney test | 0.2704 | no | N/A | N/A | normal diet, 20; ari, 19 (flies) | normal diet, 20; ari, 19 |
| S2b_nucleus | no | Kruskal-Wallis test with Dunn's multiple comparison | normal diet early N vs ari early N, ≥0.9999; normal diet early N vs. normal diet late N, ≤0.0001; normal diet early N vs ari late N, ≤0.0001; ari early N vs normal diet late N, ≤0.0001; ari early N vs. ari late N, ≤0.0001; normal diet late N vs ari late N, ≥0.9999; | normal diet early N vs ari early N, no; normal diet early N vs normal diet late N, yes; normal diet early N vs ari late N, yes; ari early N vs normal diet late N, yes; ari early N vs. ari late N, yes; normal diet late N vs. ari late N, no | N/A | N/A | normal diet early N, 114; ari early N, 92; normal diet late N, 442; ari late N, 352 (spermatogonia) | normal diet, 11; ari, 11 |
| S2b_mitoball | no | Kruskal-Wallis test with Dunn's multiple comparison | normal diet early MB vs ari early MB, ≥0.9999; normal diet early MB vs normal diet late MB, ≤0.0001; normal diet early MB vs ari late MB, ≤0.0001; ari early MB vs normal diet late MB, ≤0.0001; ari early MB vs ari late MB, ≤0.0001; normal diet late MB vs ari late MB, ≤0.0001; | normal diet early MB vs ari early MB, no; normal diet early MB vs normal diet late MB, yes; normal diet early MB vs ari late MB, yes; ari early MB vs. normal diet late MB,yes; ari early MB vs ari late MB, yes; normal diet late MB vs ari late MB, yes | N/A | N/A | normal diet early MB, 114; ari early MB, 92; normal diet late MB, 442; ari late MB, 352 (spermatogonia) | normal diet, 11; ari, 11 |
| S2c_ratio | no | Kruskal-Wallis test with Dunn's multiple comparison | normal diet early ratio vs normal diet late ratio, ≤0.0001; ari early ratio vs. ari late ratio, 0.0342; normal diet early ratio vs ari early ratio, 0.2510; normal diet late ratio vs ari late ratio, ≤0.0001 | normal diet early ratio vs normal diet late ratio, yes; ari early ratio vs. ari late ratio, yes; normal diet early ratio vs. ari early ratio, no; normal diet late ratio vs. ari late ratio, yes | N/A | N/A | normal diet early ratio, 114; ari early ratio, 92; normal diet late ratio, 442; ari late ratio, 352 (spermatogonia) | normal diet, 11; ari, 11 |
| S3b | no | Mann-Whitney test | 0.7104 | no | N/A | N/A | normal diet, 21; ari, 25 (testis) | normal diet, 11; ari, 13 |
| S3d | yes | unpaired t-test | 0.0002 | yes | normal diet, 1089; ari, 1788 | normal diet, 291.0; ari, 447.0 | normal diet, 14; ari, 16 (testis) | normal diet, 7; ari, 8 |
| S4b | no | Mann-Whitney test | ≤0.0001 | yes | N/A | N/A | normal diet, 13; ari, 21 (testis) | normal diet, 7; ari 11 |
| S4c | yes | Ordinary one-way ANOVA with Tukey's multiple comparison | normal diet vs ari, 0.0169; normal diet vs ari + LA 0.9345; ari vs ari + LA, 0.0361 | normal diet vs. ari, yes; normal diet vs ari + LA, no; ari vs ari + LA, yes | normal diet, 6.704; ari, 5.604; ari + LA, 6.450 | normal diet, 1.315; ari, 0.961; ari + LA, 1.198 | normal diet, 26; ari, 34; ari + LA, 29  (testis) | normal diet, 13; ari, 17; ari + LA, 15 |
